# Expression and DNA methylation of 20S proteasome subunits as prognostic and resistance markers in cancer

**DOI:** 10.1002/1878-0261.70038

**Published:** 2025-08-27

**Authors:** Ruba Al‐Abdulla, Simone Venz, Ruslan Al‐Ali, Martin Wendlandt, Mandy Radefeldt, Elke Krüger

**Affiliations:** ^1^ Institute of Medical Biochemistry and Molecular Biology University Medicine of Greifswald Germany; ^2^ Centogene Rostock Germany

**Keywords:** cancer, DNA methylation, patient survival, proteasome, TCGA data

## Abstract

Proteasomes are involved in the maintenance of cellular protein homeostasis and the control of numerous cellular pathways. Single proteasome genes or subunits have been identified as important players in cancer development and progression without considering the proteasome as a multisubunit protease. We here conducted a comprehensive pan‐cancer analysis encompassing transcriptional, epigenetic, mutational landscapes, pathway enrichments, and survival outcomes linked to the 20S proteasome core complex. The impact of proteasome gene expression on patient survival exhibited a cancer type‐dependent pattern. Increased proteasome expression correlated with elevated activation of oncogenic pathways, such as DNA repair, MYC‐controlled gene networks, MTORC1 signalling, oxidative phosphorylation, as well as metabolic pathways including glycolysis and fatty acid metabolism. Accordingly, potential loss of function variants of proteasome subunit genes are associated with improved patient survival. The TCGA‐derived outcomes were further supported by gene expression analysis of THP‐1 cells. Our study highlighted the importance of studying the proteasome as an enzymatic functional unit rather than separated subunits.

## Graphical abstract

Comprehensive analysis of genomic mutations, gene expression, DNA methylation, and pathway analysis of TCGA data was carried out to define cancer types in which proteasome subunits expression is associated with worse survival. Albeit the effect of specific proteasome subunits on cellular function, the main role of the proteasome is better evaluated when the proteasome is studied as an enzyme unit.

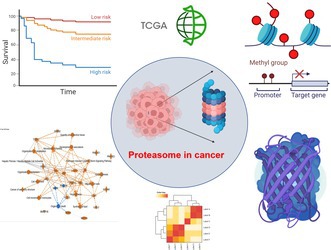

**Abbreviations**
ACMG: The American College of Medical Genetics and Genomics
BTZ: bortezomib
CP: core particle
GSEA: gene set enrichment analysis
IP: immunoproteasome
IPA: ingenuity pathway analysis
NES: normalized enrichment score
QC: quality control
RP: regulatory particle
SP: standard proteasome
TCGA: The Cancer Genome Atlas
UPR: unfolded protein response
UPS: ubiquitin proteasome system

## 1 Introduction

Protein homeostasis is crucial for cellular function and survival, it is maintained through protein synthesis and degradation balance, known as protein quality control [1]. The ubiquitin proteasome system represents the primary intracellular and nonlysosomal mechanism for protein degradation within cells [2]. The proteasome, with a conserved structure and protease activity, selectively degrades misfolded, regulatory, and signalling proteins, influencing processes like immune regulation, cell cycle, apoptosis, and DNA repair [3, 4, 5, 6, 7, 8]. Protein degradation by the proteasome is initiated by an enzymatic cascade of the ubiquitin conjugation machinery E1, E2, and E3, which mediate polyubiquitination of proteins for proteasomal degradation [3]. Polyubiquitinated proteins with lysine 48‐linked ubiquitin chains are typically degraded by the 26S proteasome complex, consisting of two subcomplexes, 20S core particle (CP), and the 19S regulatory particle (RP) [9, 10]. The 20S CP has a barrel structure made of four heptameric rings of α‐ and β‐subunits. The outer rings have seven α‐subunits (*PSMA1‐7*), while the inner rings have seven β‐subunits (*PSMB1‐7*) with active threonine sites. β1, β2, and β5 subunits in the 20S CP provide caspase‐, trypsin‐, and chymotrypsin‐like activities, respectively. The RP plays a crucial role in maintaining cellular protein homeostasis by recognizing and regulating access of target proteins to the proteolytic chamber [7, 9]. Besides the standard proteasome (SP), various isoforms such as the immunoproteasome (IP) exist. The IP is present in immune cells and can be induced by cytokines in other cell types. It incorporates inducible β1i‐, β2i‐, and β5i‐subunits (*PSMB8‐10*), enhancing proteolytic activity [3, 11]. Dysfunction and dysregulation of the proteasome has been linked to a wide range of diseases, including cancer, neurodegenerative disorders, and autoimmune diseases [12, 13, 14]. Several studies have reported alterations in the expression of single proteasome subunits in various types of cancer [15, 16, 17, 18]. Indeed, proteasome α‐subunits have been associated with the development and progression of several types of cancers, including hepatocellular carcinoma, breast cancer, lung cancer, and multiple myeloma [19, 20, 21, 22, 23]. Certain β‐subunits were as well identified as therapeutic targets in solid tumours such as hepatocellular carcinoma or kidney cancer [24, 25]. However, single subunits cannot function in selective degradation of target proteins. Therefore, there is an urgent need to evaluate the impact of proteasome expression and activity in cancer as an enzymatic unit, rather than the evaluation of the role of separated subunits. Proteasome expression in cancer is associated with oncogenic signalling pathways, such as the PI3K/Akt/mTOR, NF‐κB, Cyclin D1 signalling, interferon signalling, and Wnt‐β catenin pathways [26, 27, 28, 29, 30].

In addition, elevated proteasome expression has been associated with the development, progression, and therapeutic resistance of tumours. Therefore, targeting proteasomes via pharmacological inhibition potentiates the anticancer efficacy of other chemotherapeutic drugs [27]. Given the dysregulation of proteasome expression in cancer, SP and IP inhibitors have emerged as a promising class of cancer therapeutics [12]. The first proteasome inhibitor, bortezomib (BTZ), was approved by the FDA. This medication has shown significant efficacy in the treatment of multiple myeloma, mantle cell lymphoma, and several types of solid tumours [31, 32]. Several other proteasome inhibitors have been developed and are currently being investigated as cancer therapeutics [33].

In the current study we aimed to answer two questions: (a) In which types of cancer does the proteasome 20S expression possess a prognostic value, and (b) What are the oncogenic pathways by which high expression of proteasome has a worse impact on patient survival.

## 2 Methods

### 2.1 Survival analysis

To investigate the impact of the transcriptome levels of proteasome 20S subunits, namely, *PSMA1* (NM_002786.3), *PSMA2* (NM_002787.4), *PSMA3* (NM_002788.4), *PSMA4* (NM_002789.4), *PSMA5* (NM_002790.3), *PSMA6* (NM_002791.1), *PSMA7* (NM_002792.3), *PSMB1* (NM_002793.3), *PSMB2* (NM_002794.4), *PSMB3* (NM_002795.2), *PSMB4* (NM_002796.2), *PSMB5* (NM_002797.3), *PSMB6* (NM_002798.2), *PSMB7* (NM_002799.3) as well as *POMP* (NM_015932.5), on patients’ overall survival, we performed a survival analysis using Kaplan–Meier (K‐M) survival curves obtained from the TCGA portal. The median expression level of each gene was used as a cutoff value. Only cancer types with a sample number that exceeds 150 were included in the current study. TCGA abbreviations and the number of samples used are in Table S1. Our findings were confirmed using the K‐M‐plotter tool (https://kmplot.com/analysis/). The results are presented in a color‐coded table, where red indicates that elevated gene expression is associated with worse prognosis, blue indicates that high expression is associated with better survival, and pale yellow indicates that the association was not significant. We considered the results statistically significant if the Log‐rank *P*‐value was < 0.05. Cancer types were included in the study if elevated expression of 15 genes or more of the proteasome subunits showed a negative prognosis. Protein encoded by the proteasome subunits genes are listed in Table S2. The results were confirmed using Cox regression in the types of cancer included in the current study.

### 2.2 The effect of DNA methylation on proteasome expression and patient survival

In this study we investigated the impact of DNA methylation on the overall survival of patients in five types of cancer (Kidney renal clear cell carcinoma (KIRC), Acute Myeloid Leukaemia (LAML), Low‐Grade Gliomas (LGG), Liver Hepatocellular Carcinoma (LIHC), and Lung adenocarcinoma (LUAD)) by analysing the DNA methylation status of 20S proteasome subunits. To achieve this aim, survival curves for each CpG probe located in these genes were obtained from the publicly available MethSurv database (https://biit.cs.ut.ee/methsurv/). DNA methylation values are represented as beta values, which ranged from 0 to 1, and the median was used as a cutoff value [34]. The results were considered statistically significant if consistent results were obtained with a Log‐rank *P*‐value < 0.05. To present the results, we used a color‐coded table, where blue indicates that high levels of DNA methylation are associated with better patient survival, and red indicates that high levels of DNA methylation are associated with worse survival. Data of DNA methylation were downloaded, and the correlation between DNA methylation and RNA expression was determined using an R‐based code for the Spearman factor.

In addition, we examined the methylation status of the expression and activity of proteasomal subunits of our focus. THP‐1 cells were cultivated and treated with 100 nm DNA methyltransferase inhibitor (decitabine; DAC) for 7 and 14 days.

RNA was isolated utilizing the innuPREP RNA Mini Kit 2.0 (iST Innuscreen GmbH, Berlin, Germany). Subsequently, a quantitative Polymerase Chain Reaction (qPCR) was performed using the TB Green™ Premix Ex Taq™ (TaKaRa Bio Inc., Kusatsu, Shiga, Japan). Both procedures were conducted according to the manufacturer's instructions. The primer sequences employed for the qPCR are provided in Table S3. Proteasome activity and the protein expression of catalytic subunits of the proteasome were measured as previously described [35].

### 2.3 Variant interpretation of TCGA somatic sequencing data

Somatic mutations associated with proteasome 20S subunit genes were extracted from the c‐bioportal (TCGA/Pancancer Atlas dataset), which included data from five different types of cancer. Variants were preselected using the aggregator and impact analysis tool VarSome [36] based on factors such as predicted consequence, structural and functional impact, and phylogenetic conservation. Subsequently, the resulting variants were then manually classified following the updated American College of Medical Genetics and Genomics (ACMG) criteria [37]. Nomenclature for genetic information adheres to the recommendations provided by the Human Genome Variation Society (HGVS) [38]. For structural analysis, mature 20S subunit protein sequences containing a substitution of interest were subjected to folding using AlphaFold. Subsequently, these folded structures were compared with their corresponding reference structures in the context of the complete 20S proteasome core particle obtained from PDB ID 5LF3, using chimerax version 1.6.1. This comparison also allowed for the visualization of hydrogen bonds, electrostatic potential, and hydrophobicity.

All variants extracted from the KIRC, LAML, LGG, LIHC, and LUAD samples from the TCGA database underwent dbSNFP4.4 variant effect prediction via the VarSome web application. The cutoff values adhered to the recommendations of VarSome's ‘Calibrated In‐Silico Thresholds’ (calibrated on 02/Dec/2022) that follow Clinical Genome Resource (ClinGen) guidelines [39]. Here, we excluded synonymous and intronic variants where no splice relevance was indicated and raw‐filtered the remaining variants for an overall positive aggregated meta predictor score. In a subsequent step, these variants were assessed in greater detail according to their MetaRNN and BayesDel (addAF) for missense variants, or scSNV‐ADA scores for potential splice variants, respectively. Additionally, structural properties such as functional domains, secondary structures, or possible modifications found in the Uniprot data, in conjunction with evolutionary model‐based single predictors CADD and PhyloP, were incorporated into the analysis. This process yielded the 16 variants of “strict” interest (VOIs) described in our publication that were manually classified using the ACMG criteria (Table S4). The correlation between overall and proteasome mutations and Kaplan–Meier survival is shown in Table S5.

### 2.4 Pathway analysis

The pathway analysis was determined using the gene expression files downloaded from c‐bioportal‐TCGA/Pancancer dataset. Data were then stratified based on the expression of both *PSMAs* (1–7), *PSMBs* (1–7), and *POMP*. High expression was defined as the expression of at least 10 out of the 15 genes above the median; otherwise, it was classified as a sample with low expression. This stratification was carried out using the Anaconda‐Jupyter python code, and followed by a pathway analysis using the gene set enrichment analysis (GSEA) [40, 41]. We applied the next parameters (1000 repeat, Reactome, and hallmark pathway studies) using a cutoff *P*‐value of 0.05 with a false discovery rate (FDR) value of 0.25. For the sake of simplicity, we only show the results of the hallmark platform. Confirmation of the results was carried out with a second strategy of stratification using the mean of the 15 genes and apply the median of this mean as a cutoff value afterwards (data provided upon request).

Gene ontology, functional analyses, as well as pathway network analyses were performed with the expression data using the Ingenuity Pathways Analysis tool (IPA, QIAGEN Bioinformatics, Redwood, CA, USA; https://www.qiagenbioinformatics.com/products/ingenuity‐pathway‐analysis/). The *z*‐score algorithm was employed to forecast the direction of alteration in the present activity. IPA's *z*‐score is a measure that forecasts the activation or inhibition of a pathway or gene. A negative *z*‐value (blue) suggests that the pathway is mostly inhibited, while a positive *z*‐value (orange) indicates that the pathway is predominantly activated. According to company descriptions (https://qiagen.my.salesforce‐sites.com/KnowledgeBase/KnowledgeNavigatorPage?id=kA41i000000L5pACAScategoryName=IPA) the canonical pathways analysis exhibits the most noteworthy pathways in the complete dataset, with significance determined by the right‐tailed Fisher's Exact *T*‐test, indicating the likelihood of association between molecules from the dataset and the canonical pathway occurring randomly. The data from the analysis were visualized as a bubble chart, in which the *y*‐axis shows the pathway categories, and the *x*‐axis shows their *z*‐score. The pathway bubbles are coloured based on their *z*‐score and the bubble size is proportional to the number of genes from the dataset that overlap with each pathway.

### 2.5 Cell culture and RNA‐seq analysis

THP‐1 (RRID: CVCL_0006) cells were cultivated in RPMI medium supplemented with foetal calf serum (FCS) 10% and penicillin/streptomycin and incubated at 37 °C and CO_2_ 5%. An authentication test was carried out using genomic DNA to prove the identity of the cells applying Short Tandem Repeat analysis (STR). In addition, cells were routinely checked for mycoplasma using polymerase chain reaction (PCR). All experiments were carried out in mycoplasma‐free cells. Cells were treated with bortezomib at a nontoxic dose (10 nm) for 16 h. After collection, RNA was extracted and sequenced at Centogene GmbH‐Ro stock. RNA quality was assessed with the RNA Screentape on the Agilent Tapestation (Agilent, Santa Clara, CA, USA).

#### 2.5.1 RNA sequencing

RNA libraries were prepared using the Illumina stranded mRNA kit (Illumina, San Diego, CA, USA). Quality Control (QC) of the libraries was conducted with the DNA 1000 Screentape on an Agilent Tapestation. Samples were sequenced on an Illumina NextSeq500 using the NextSeq 500/550 High Output v2.5 reagents and the 75 bp paired‐end protocol. RNA‐seq raw data was aligned using the two‐pass mode with star v.2.7.6a against hg19 [42]. The read groups are fixed, and duplicates marked using picard tools v.2.23.8 (http://broadinstitute.github.io/picard). Counting the reads was performed by feature counts/subread v.2.0 [43]. Pathway enrichment analysis was performed with Gene Set Enrichment Analysis (GSEA) gene differential analysis [40]. Gene differential analysis was further confirmed with deseq2 [44] and toppgene [45].

### 2.6 Correlation study

The correlation between the gene expression of 20S proteasome subunits and specific genes was carried out using GEPIA2 [46]. The Pearson factor was used to estimate the correlation. The gene signature of the proteasome 20S subunits was created using the 15 genes included in this study. The gene signature of ABC transporters was created using 24 genes of the ABC family of transporters (*ABCA1*, *ABCA2*, *ABCA3*, *ABCA5*, *ABCA7*, *ABCB10*, *ABCB4*, *ABCB6*, *ABCB7*, *ABCB8*, *ABCB9*, *ABCC1*, *ABCC10*, *ABCC4*, *ABCC5*, *ABCC6*, *ABCD1*, *ABCD3*, *ABCD4*, *ABCE1*, *ABCF1*, *ABCF2*, *ABCF3*, and *ABCG1*). These genes were chosen due to the fact that they are expressed in THP‐1 cells, which facilitates the validation of the obtained data. A correlation was considered poor if the *R* value was below 0.2, fair if *R* the value was between 0.3 and 0.5, and moderate if the *R* value was between 0.6 and 0.8. A very strong correlation was observed when the *R* value was above 0.8 [47]. The correlation between gene expression and DNA methylation probes was carried out using data obtained from MethSurv. The Spearman factor was calculated for each CpG probe and the corresponding gene. Results were considered significant if *P* < 0.05. Original data tables are provided in Data S1.

## 3 Results

### 3.1 Correlation between proteasome expression and survival of patients with cancer

**Fig. 1:**
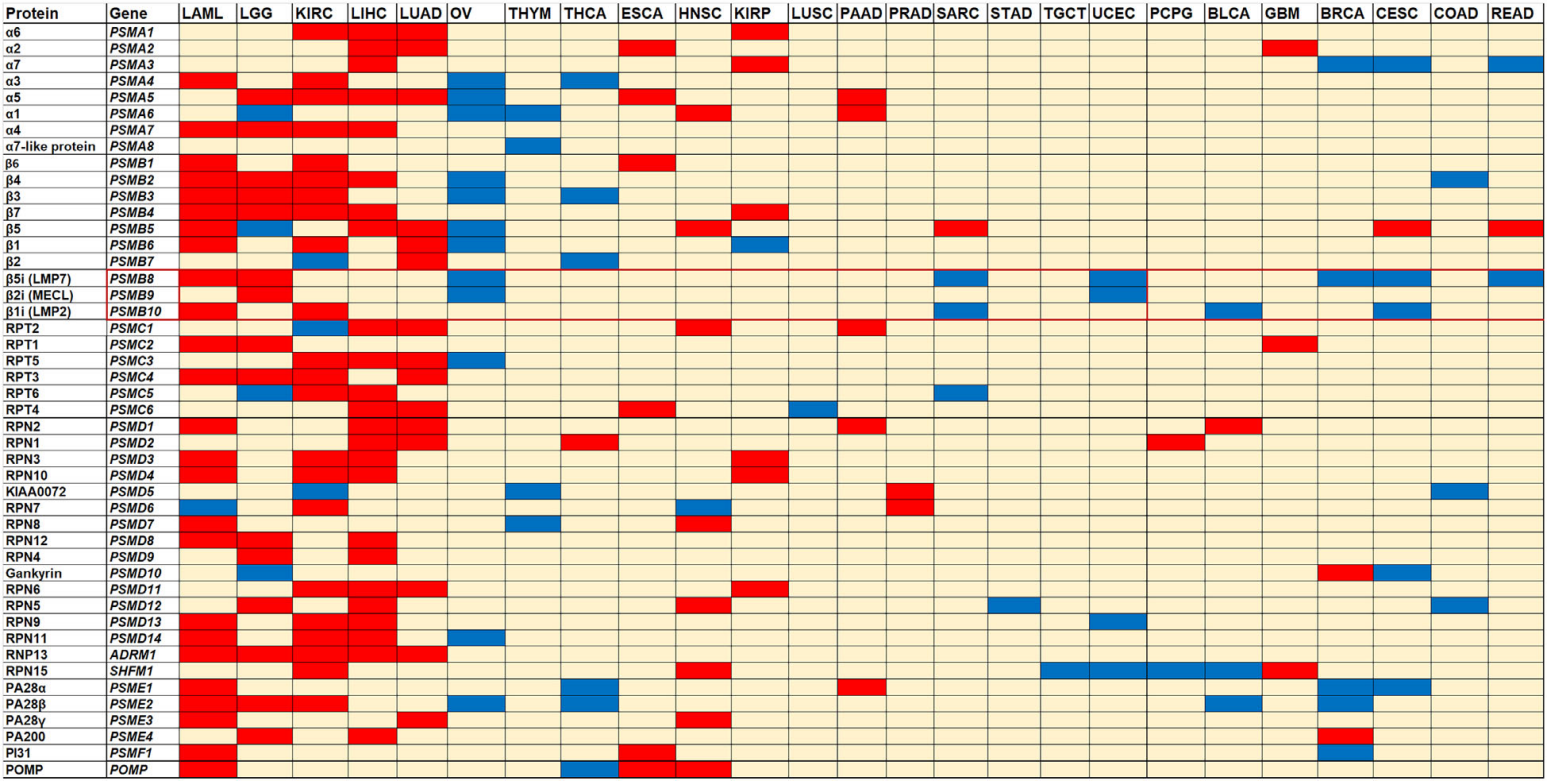
Association between proteasome subunit expression and overall survival in patients with different types of cancers. Kaplan–Meier survival curve for each gene is obtained in each type of cancer from TCGA PORTAL (abbreviation and sample number are provided in Table S2). Median is used as a cutoff value and *P* value is calculated using the log‐rank test. Difference is considered significant if *P* ≤ 0.05. Results for association of high expression and survival are presented in colour code: Red – worse overall survival; Blue – better overall survival; pale Yellow – no significant association.

First, we aimed to assess the prognostic value of proteasome subunit expression in different cancer types available in the cancer genome atlas (TCGA) database by using Kaplan–Meier survival curves. Our findings revealed a distinct cancer type‐dependent pattern of association between the genetic expression of proteasome subunits and patient survival (Fig. 1). Table S1 provides the abbreviations for these cancer types along with the number of patient samples analysed in each category. Additionally, the abbreviations for proteasome genes and their corresponding proteins are presented in Table S2.

In the case of acute myeloid leukaemia (LAML) and hepatocellular carcinoma (LIHC), high expression of proteasome subunits was predominantly associated with poorer survival outcomes. Conversely, the pattern observed in other tissues such as the brain, kidney, and lung varied depending on the cancer type. Notably, proteasome subunits expression did not significantly impact patient survival in glioblastoma multiforme (GBM), kidney renal papillary cell carcinoma (KIRP), and lung squamous cell carcinoma (LUSC). However, in the same organs, for low‐grade glioma (LGG), kidney renal cell carcinoma (KIRC), and lung adenocarcinoma (LUAD), high expression of proteasome subunits genes was linked to worse patient survival. On the other hand, ovarian serous cystadenocarcinoma (OV) and, to a lesser extent, thyroid carcinoma (THCA) demonstrated a favourable prognostic value of proteasome subunits expression. In these cases, elevated expression of proteasome subunits was associated with improved overall survival. Specifically, the expression levels of proteasome subunits in adrenal cancer, breast cancer, oesophageal cancer, stomach adenocarcinoma, colon cancer, pancreatic cancer, prostate cancer, thymus cancer, testicular cancer, cervical cancer, and uterine cancer did not exhibit a significant association with altered patient survival. The IP subunits (*PSMB8‐10*) exhibited a distinct pattern compared to the standard proteasome. While a worse prognosis was primarily observed in LAML and LGG, enhanced patient survival was noted in OV, uterine corpus endometrial carcinoma (USEC), cervical squamous cell carcinoma and endocervical adenocarcinoma (CESC), and sarcoma (SARC) (Fig. 1). For the sake of simplicity, we focused our further analysis on the genes encoding the SP 20S subunit, which encompass *PSMA1‐7*, *PSMB1‐7*, and the proteasome assembly protein *POMP*. The most relevant results where high proteasome 20S expression is associated with worse survival was observed in LAML, LGG, LIHC, LUAD, and KIRC. Therefore, we restricted our further analysis to these types of tumours. These results were confirmed also using Cox‐regression analysis (Data S1).

### 3.2 Gene expression, DNA methylation of proteasome 20S subunit genes, and overall survival of patients in cancer

**Fig. 2:**
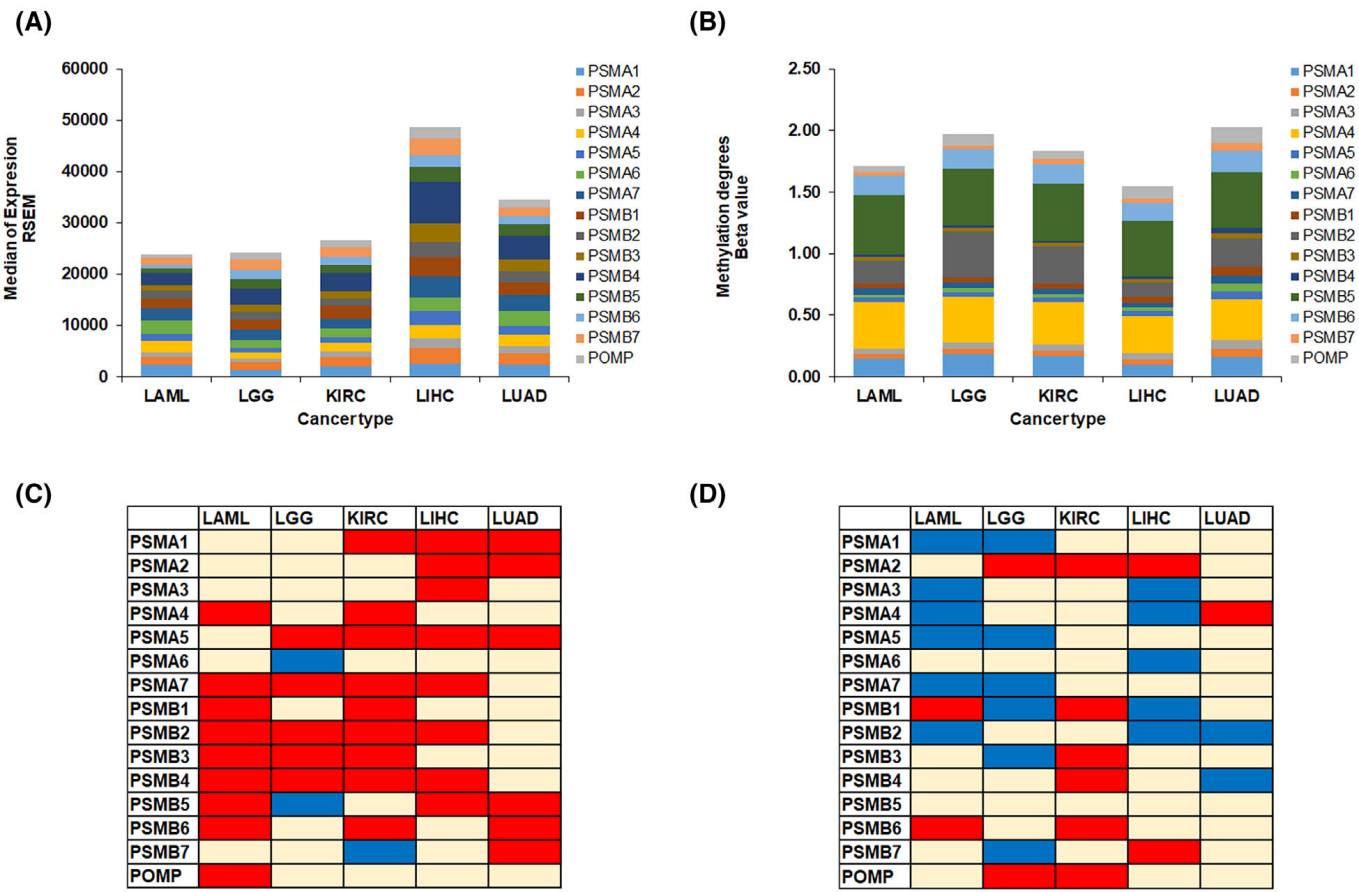
Gene expression, DNA methylation of proteasome 20S subunit genes, and patient survival in cancer for LAML, LGG, KIRC, LIHC, and LUAD. (A) Gene expression of proteasome 20S subunit genes calculated as the median of RSEM in TCGA samples (LAML: *n* = 151, LGG: *n* = 511, KIRC: *n* = 538, LIHC: *n* = 371, LUAD: *n* = 533). (B) DNA methylation of the abovementioned genes represented as beta value for the probes of the promoter obtained from UALCAN database. (C, D) colour‐based table of Kaplan–Meier survival of cancer patients based on the expression (C) or DNA methylation (D) of proteasome subunits. Association of high gene expression/DNA methylation with better survival (coloured in blue) and worse survival (coloured in red), and the absence of significant differences was coloured in pale yellow. Median was used as a cutoff value. Log‐rank test is used for the survival analysis and differences were considered significant when *P* ≤ 0.05.

It is well established that DNA methylation is involved in the regulation of gene expression, and such a regulation is disturbed in cancer [48]. In the current study we aimed to investigate if hypomethylation of the proteasome 20S subunits is associated with worse overall survival of cancer patients. Therefore, further analysis was conducted using TCGA data to evaluate the association between DNA methylation of the 20S proteasome subunits and overall survival of patients. We evaluated gene expression (obtained from c‐bioportal) and DNA methylation (obtained from UALCAN‐TCGA) in LIHC, LAML, LGG, LUAD, and KIRC. The expression of proteasome subunits was found to be abundant in cancer (Fig. 2A). The most elevated levels of expression are seen in LIHC, which was associated with lower methylation levels (Fig. 2B). We observed higher expression of *PSMB1*, *PSMB3*, and *PSMB4* in tumour samples when compared to other subunits (Fig. 2A). Moreover, elevated expression in the tumour was observed when compared with nontumor surrounding tissue in LIHC, LUAD, and KIRC for almost all proteasome subunits. The highest levels of DNA methylation were observed in *PSMA4* and *PSMB5* (Fig. 2B). The levels of DNA methylation of *PSMB2* were higher in LUAD, KIRC, and LGG, and lower DNA methylation of this subunits was observed in LAML and LIHC (Fig. 2B). Lower levels of DNA methylation of the proteasome 20S subunits were observed in tumour samples when compared to nontumour surrounding tissue in LIHC and LUAD. However, in KIRC, a slight elevated DNA methylation was observed in tumour samples when compared with nontumour tissue. Indeed, our results indicate that elevated expression and hypomethylation of the proteasome 20S subunits is associated with worse survival in LIHC, LGG, and LAML (Fig. 2C,D).

**Fig. 3:**
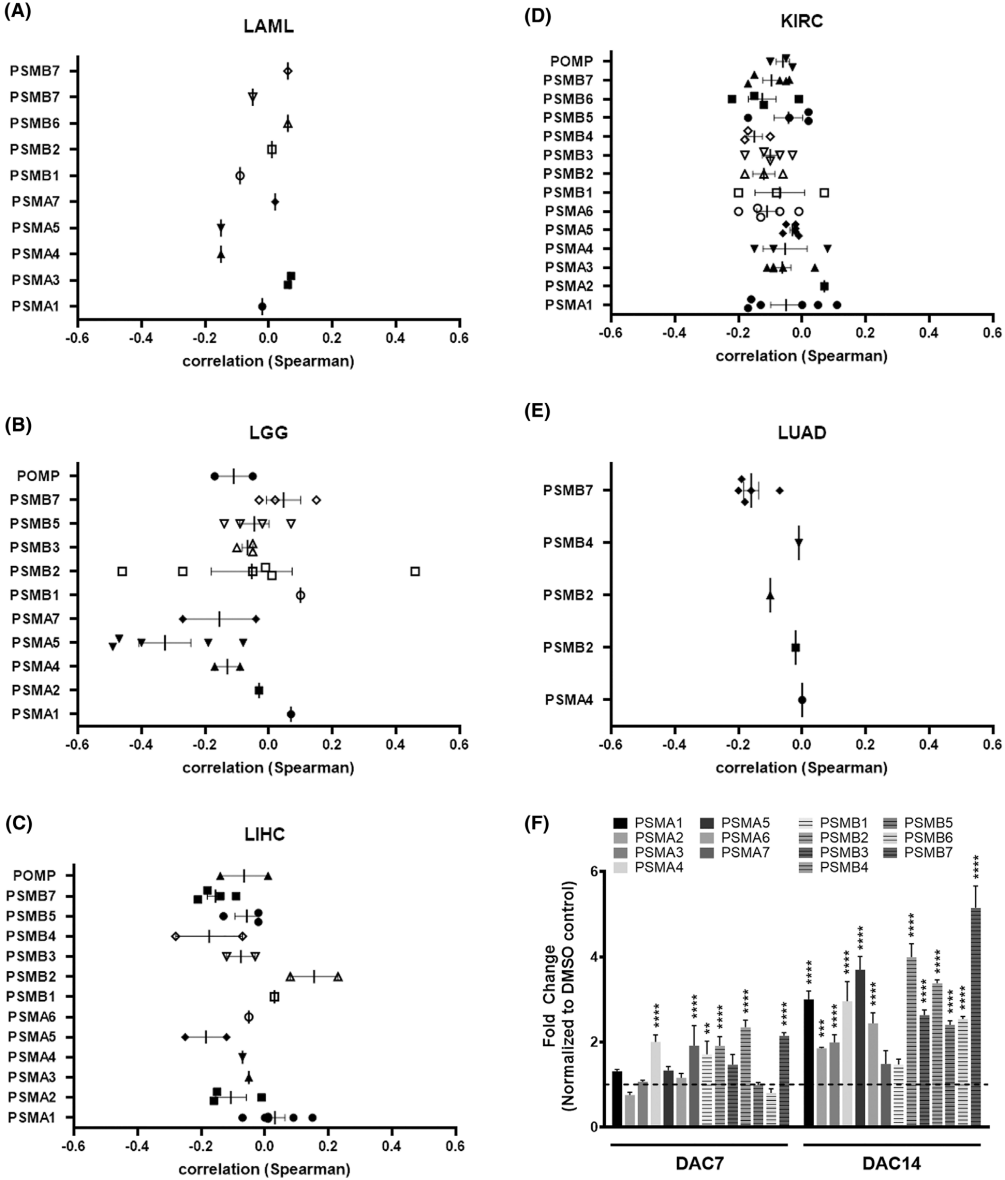
Correlation between DNA methylation and expression of proteasome 20S subunit genes. Box blot of Spearman correlation factor of CpGs probes and gene expression of each gene in (A) LAML, *n* = 151; (B) LGG, *n* = 511; (C) LIHC, *n* = 371; (D) KIRC, *n* = 538; (E) LUAD, *n* = 533; (F) Differentially expression of proteasome subunits in THP‐1 cells after treatment with 100 nm of DNA methyltransferase inhibitor (decitabine; DAC) for 7 (DAC7) or 14 days (DAC14), *n* = 3. DNA methylation data (β‐value for each probe) and gene expression data (RSEM) were obtained from metsurv TCGA pan‐cancer data. Each point represents a correlation of specific DNA methylation probe with gene expression of the corresponding gene. Gene expression data are presented as mean ± SD. ANOVA one‐way was used for statistical analysis. *P ≤ 0.05.

The correlation between DNA methylation and gene expression of each subunit was then carried out using the Spearman factor. In LGG (Fig. 3B), a significant negative correlation was observed between the expression of *PSMA5* and *PSMB2* and the methylation degree of several probes that are located in these genes, which indicates that DNA methylation could be a mechanism by which the expression of these genes is regulated. Similar results were obtained for the DNA methylation of *PSMA5* and *PSMB4* in LIHC (Fig. 3C). No marked negative correlation was observed between the expression of proteasome 20S subunit genes and DNA methylation at the corresponding probes in LAML, KIRC, and LUAD, as observed in Fig. 3A,D,E, respectively. To mimic hypomethylation, we treated THP‐1 cells with the DNA methyltransferase inhibitor (decitabine; DAC) for 7 or 14 days and measured gene expression of proteasome subunits. Indeed, almost all 20S subunit genes were significantly upregulated in response to DAC treatment (Fig. 3F).

### 3.3 Variant identification of TCGA somatic sequencing data

**Fig. 4:**
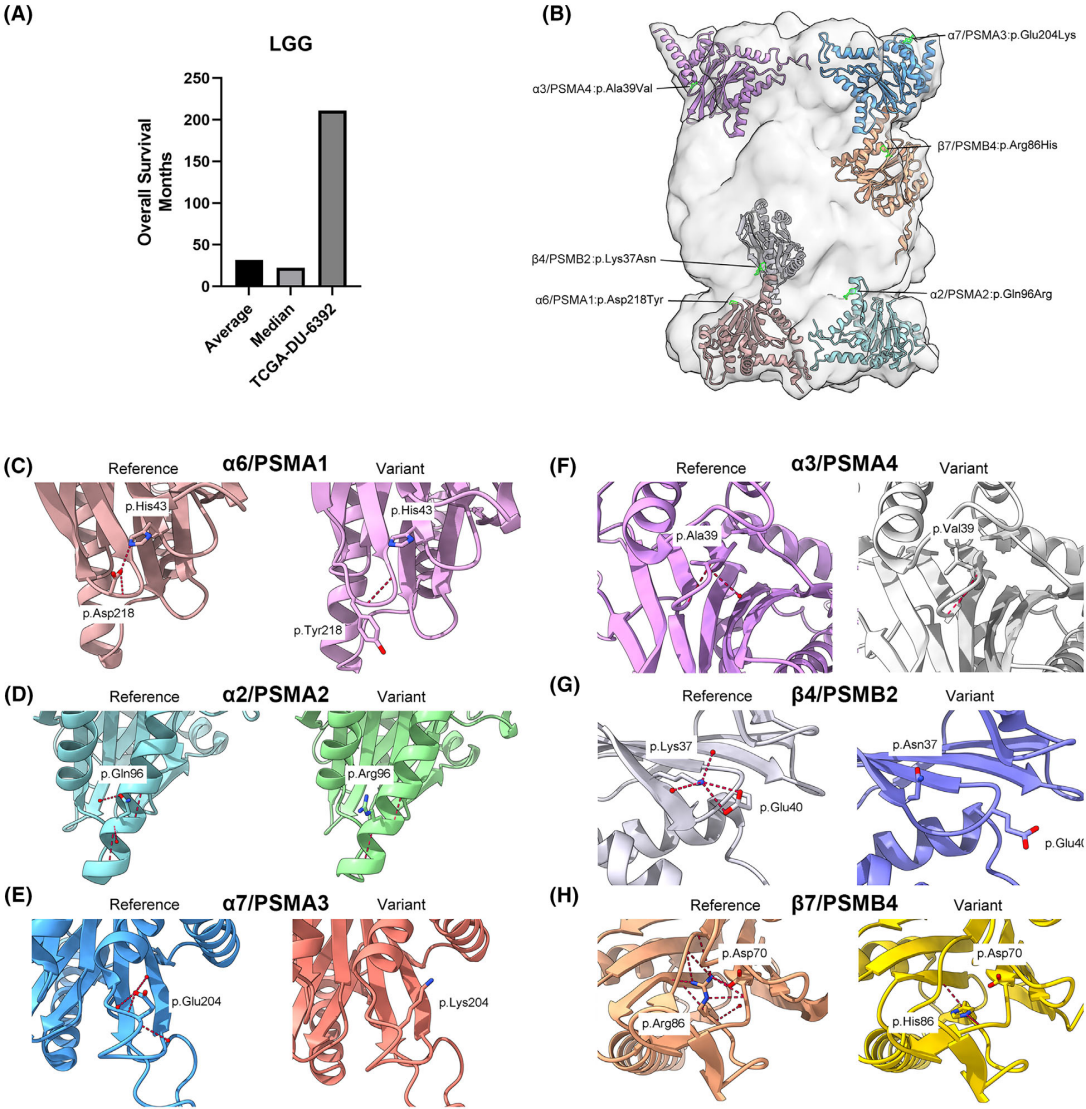
Survival and structural representation of the variants found in sample TCGA‐DU‐6392. (A) Survival of TCGA‐DU‐6392 compared to the average and median LGG (*n* = 511) survival in months. (B) Location of the six standard core particle variants within each respective subunit. (C–H) Comparison between each subunit's reference structure (left) and the corresponding folded variant structure (right) of this sample. Red dotted lines represent hydrogen bonds, and residues that are putatively interacting with the identified variants are also illustrated.

Elevated gene expression, and DNA hypomethylation of the 20S proteasome subunits was found to be associated with worse overall survival in patients with LAML, LIHC, KIRC, LGG, and LUAD. To study if loss of function of the enzymatic activity of the proteasome is in turn associated with improved survival, we studied a specific LGG case in which multimutations in different proteasome subunits were detected. A total of 112 variants were identified by extracting data from the TCGA dataset for the 15 proteasome‐associated genes and narrowed down to 16 variants of interest after the preselection process using VarSome and ACMG criteria. Among them, we discovered six likely pathogenic variants (ACMG Class 4) and 10 variants of uncertain significance (ACMG Class 3) that exhibited a stronger indication of a potential functional impact. Specifically, these variants consisted of three frameshift variants, four canonical splice variants, and nine missense variants, as summarized in Table S6. All identified variants were absent in normal tissue samples and occurred at frequencies ranging from 0.06 to 0.69. One sample (TCGA‐DU‐6392) with the variant p.Arg86His in β7/PSMB4 stood out in particular. This patient, diagnosed with LGG, demonstrated an exceptionally extended survival period, surpassing the median survival by almost 10‐fold (Fig. 4A). Upon further investigation, it became evident that this particular sample harboured the highest number of 20S subunit variants and six of them being located in the SP core particle (Fig. 4B–H): α6/PSMA1:p.Asp218Tyr, α2/PSMA2:p.Gln96Arg, α7/PSMA3:p.Glu204Lys, α3/PSMA4:p.Ala39Val, β4/PSMB2:p.Lys37Asn, β7/PSMB4:p.Arg86His. All variants were exclusive to the patient's tumour samples and not present in the normal tissue. According to the predictions, the β7/PSMB4 and the α7/PSMA3 variant, with a somatic frequency of 0.44 and 0.30, respectively, are expected to exert the most profound consequences on proteasome structure and function (Fig. 4C–H and Fig. S1A–F).

In the β7/PSMB4 reference structure, p.Arg86 is anticipated to interact with an upstream aspartate at position 70, possibly in conjunction with an arginine at position 227 (not shown), forming stabilizing hydrogen bonds. However, in TCGA‐DU‐6392, these hydrogen bonds are disrupted by the histidine substitution at position 86, likely resulting in the destabilization of this region (Fig. 4H). This region serves as a contact area with the adjacent β1/PSMB6 subunit, apart from the β7/PSMB4 C‐terminal interaction with the β1/PSMB6 catalytic domain of the opposite β‐ring.

The glutamate at position 204 in α7/PSMA3 is a component of a turn that establishes contact with the 19S particle, particularly the Rpt3/PSMC4 subunit. The significant change from the negatively charged glutamate to the positively charged lysine at this position in the sample is expected to induce destabilization in this region and disrupt the interaction with the 19S particle (Fig. S1C). These results indicate a possible association between prolonged survival and reduced proteasome function.

To determine whether the observed increase in survival was due to the overall number of mutations or specifically attributable to the number of proteasome mutations, we calculated the Spearman correlation for the overall number of mutations and the Kaplan–Meier survival for both the LGG and the complete TCGA dataset. Subsequently, we calculated the correlation between proteasome mutations and the Kaplan–Meier survival estimates of the complete TCGA dataset to assess the impact of proteasome mutations. No correlation was found between the overall number of mutations and improved survival (with ρ values of −0.07 and −0.05, respectively). Conversely, a poor correlation of ρ = 0.16 was observed between the number of proteasome mutations and patient survival (Table S5).

### 3.4 Pathway enrichment in patients with high and low 20S proteasome subunit expression

**Fig. 5:**
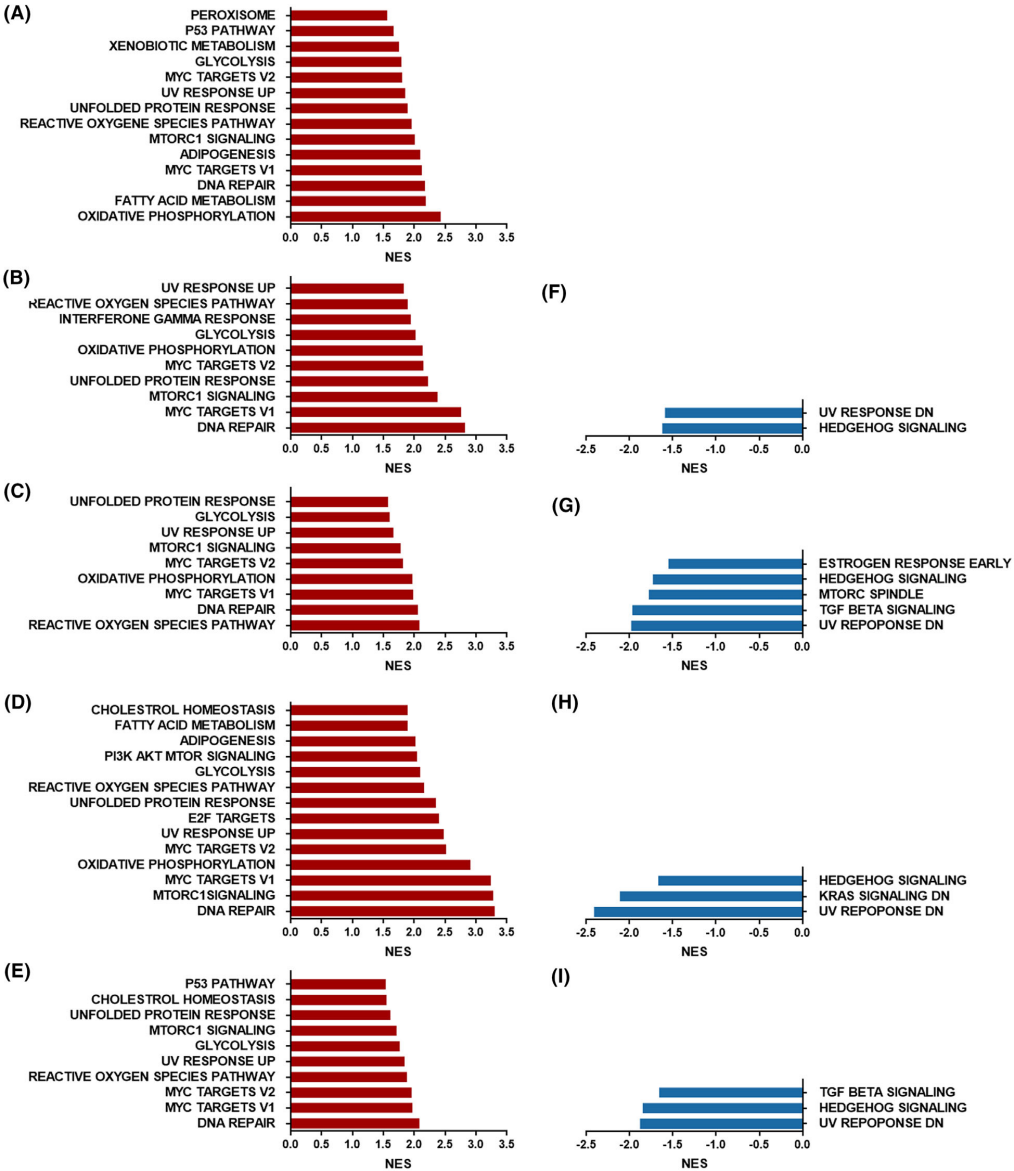
Pathway enrichment of five different cancer types given by GSEA analysis. Pathways enriched in samples with high expression of proteasome (A–E) or low expression of proteasome subunits (F–I) are shown in bar plots (in red for pathways enriched when proteasome is highly expressed and blue for pathways that associates with lower proteasome expression). Normalized enriched scores (NES) were obtained from GSEA analysis. Significant enrichment was considered if *P* ≤ 0.05 and FDR ≤ 0.25 (*P* value is provided by the software and derived from empirical permutation testing). (A) data for LAML, *n* = 151; (B, F) data for LGG, *n* = 511; (C, G) data for LIHC, *n* = 371; (D, H) data for LUAD, *n* = 533, and (E, I) data for KIRC, *n* = 538.

To estimate the functional role of 20S proteasome subunits function in cancer, we carried out a pathway enrichment analysis in the five types of cancer included in the current study. Patients’ samples were stratified based on the expression level of the median of proteasome subunits (high *vs* low). Pathway enrichment analysis is then carried out using the gsea software applied using the hallmark platform (Fig. 5). The results are presented as blot bars in red (enriched in the high‐expression group) and blue (enriched in the low‐expression group). We found elevated expression of several pathways in all studied types of cancer (Fig. 5A–E). These pathways include DNA repair, MYC targets, MTORC1 signalling, oxidative phosphorylation, reactive oxygen species, and metabolic pathways such as glycolysis and fatty acid metabolism. In the low‐expression group, except for LAML, a significant enrichment was observed in the Hedgehog signalling, pointing to dysregulated stem cell differentiation [49]. While the upstream part of the UV response was enriched in the high‐expression group, the downstream part of this pathway was enriched in the low‐expression group. Enrichment of the TGF‐β pathway was observed in the low‐expression group of LIHC and KIRC (Fig. 5G,I, respectively). To validate the results, the analysis was repeated using the mean of each sample for the 15 genes with a median cutoff value for stratifications. With this method, we obtained similar data. Elevated expression of ABC transporters is known to be associated with elevated resistance in cancer [50]. When the Reactome platform was used to carry out this analysis, a significant enrichment in the ABC transporter, stem cells markers, and RNA‐binding proteins were observed in the group with high proteasome expression.

### 3.5 Ingenuity pathway analysis of proteasome expression in cancer

The selected cancers KIRC, LAML, LGG, LIHC, and LUAD were now classified based on changes in 14 proteasomal subunits and the proteasome maturation protein POMP using ingenuity pathway analysis (IPA). For this purpose, the intensities of gene expression of cancer type associated patients were used to divide patients into two cohorts (high and low). Differentially expression values are available in Table S7. According to the classification into the respective cohorts, a comparison of gene expression between high and low was performed for all genes included in the dataset. A cutoff of 1.5 was selected for the ingenuity pathway analysis (IPA). Table S8 shows the numbers of differentially expressed genes used in the approach.

The most important pathway categories are cellular immune response, generation of precursor metabolites and energy, cellular growth, proliferation, and development, as well as neurotransmitter and other nervous system signalling (Table 1).

**Table 1:** *z*‐Scores for canonical pathways. The significant influenced pathways from IPA analysis were given by the most increased or decreases *z*‐scores.

Category	KIRC	LAML	LGG	LIHC	LUAD
Cellular immune response	< −4	< −3.5	> 4.5		< −4.5
Cellular stress and injury			> 4.5		
Generation of precursor metabolites and energy	> 7	> 5.5		> 7	> 7
Cellular growth, proliferation and development			< −8	< −7	
Neurotransmitter and other nervous system signalling			< −8	< −7	

**Fig. 6:**
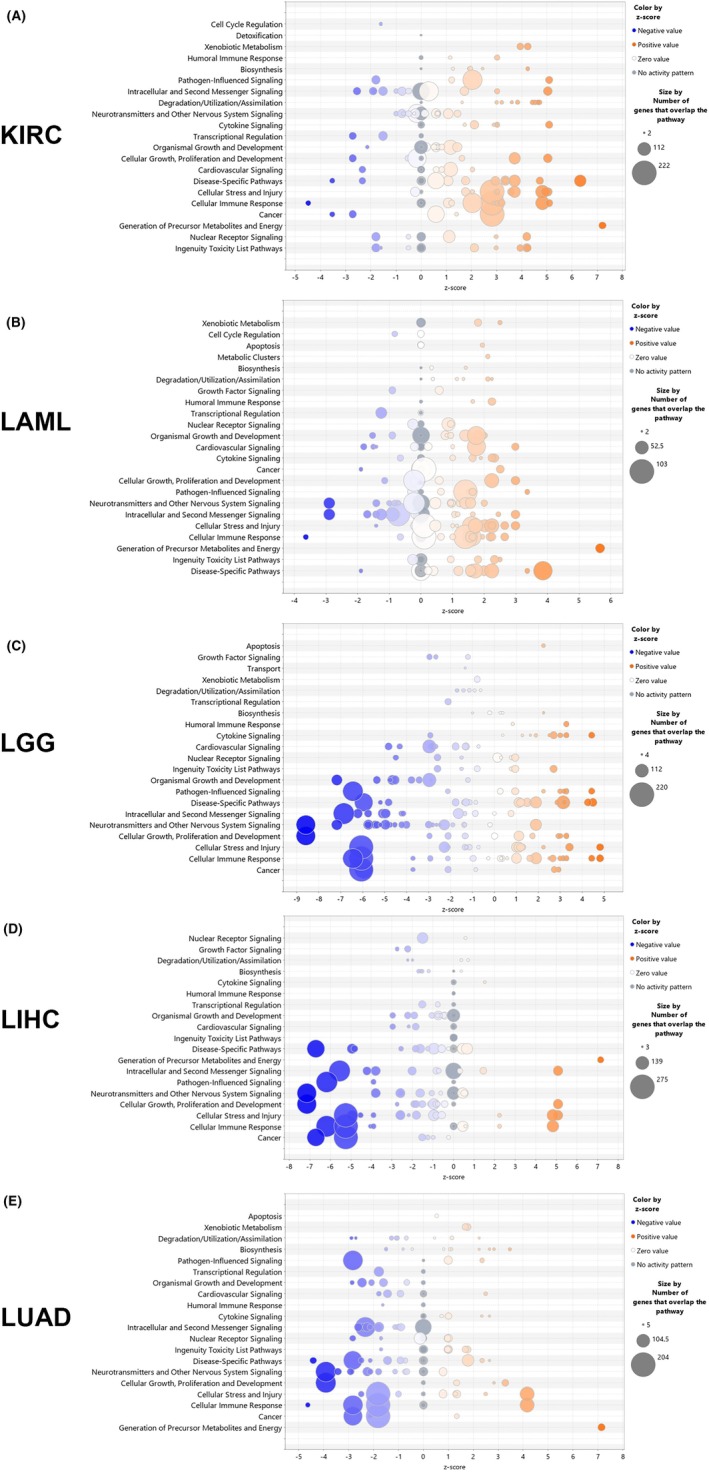
Canonical pathways for five different cancer types given by the IPA analysis. The comparison of differentially expressed genes fitted to canonical pathway were shown as bubble chart plots; pathway categories (*y*‐axis) versus the *z*‐score (*x*‐axis). The bubbles were coloured by *z*‐score (blue‐negative value; orange‐positive value) and bubble size correlates to the number of analysis‐ready genes from the dataset that overlap each pathway. (A) KIRC, *n* = 538; (B) LAML, *n* = 151; (C) LGG, *n* = 511; (D) LIHC, *n* = 371; (E) LUAD, *n* = 533.

The IPA analysis resulted in the canonical pathways shown in Fig. 6 for the respective cancer type as a bubble chart. For KIRC (Fig. 6A) and LAML (Fig. 6B), more of the genes showed an influence for the activation of pathways, whereas in LGG, LIHC, and LUAD (Fig. 6C–E), more genes were annotated to the inhibition of pathways. Additional information is given in the graphical summary of the analysis, including entities such as canonical pathways, upstream regulators, diseases, and biological functions. The overview illustrates how those concepts relate to one another (comprehensive synopsis, Fig. S2).

### 3.6 Pathway enrichment in THP‐1 cells treated with proteasome inhibitor bortezomib (BTZ)

**Fig. 7:**
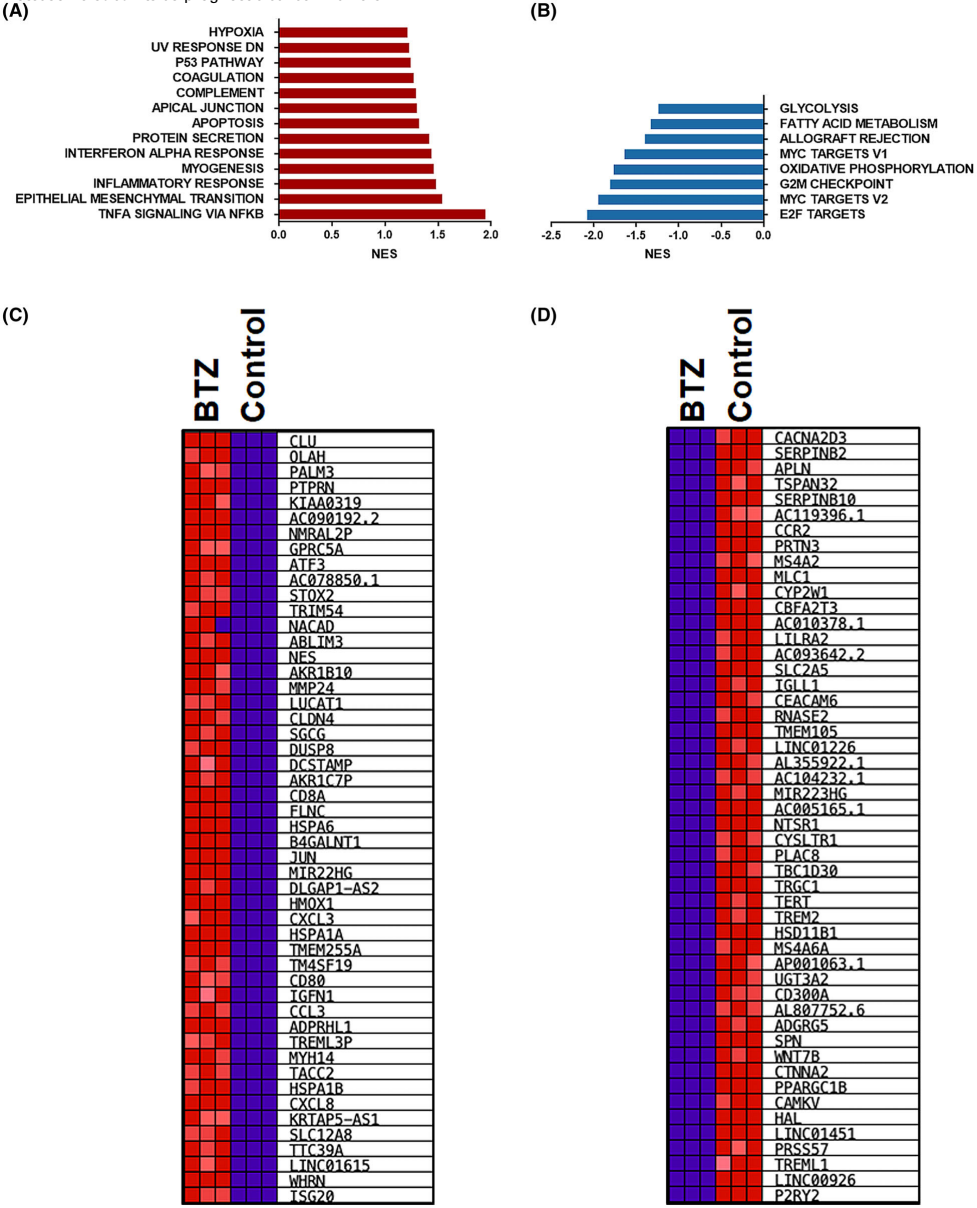
Pathway enrichment analysis and heatmap in cancer cells treated with proteasome inhibitor. RNA‐seq followed by pathway analysis was carried out for THP‐1 cells after exposure to nontoxic dose (10 nm) of bortezomib, BTZ for 16 h using GSEA analysis; the results are presented as normalized enrichment score (NES). Pathway was considered enriched if *P* ≤ 0.05 and FDR ≤ 0.25. (*P* value is provided by the software and derived from empirical permutation testing). (A) enriched pathways in treated cells compared to the control; (B) enriched pathways in control compared to the treated cells; (C) Top 50 genes that show the most elevated expression in treated cells; (D) Top 50 genes that are downregulated genes in treated cells.

As we have noticed that higher proteasome expression is associated with worse survival via the modification of oncogenic signalling pathways, we decided to confirm these changes on a functional level in cells treated with the proteasome inhibitor bortezomib (BTZ). For validation, we used THP‐1 as a cancer cell model for LAML. After treatment with a nontoxic dose of BTZ, RNA‐seq followed by pathway enrichment analysis was carried out using GSEA. Proteasome inhibition caused enrichment in the apoptosis pathway as well as the p53 pathway. It also induced immune response‐related pathways such as NF‐κB, interferon α response, and an inflammatory response (Fig. 7A). These pathways are known to sensitize cancer cells to immune therapy and enhance immunogenic cell death. As expected, on the metabolic level, while enrichment in the fatty acid metabolism and glycolysis was associated with high expression of proteasome subunits, we found that these pathways were downregulated after the proteasome inhibition using BTZ (Fig. 7B). The most inhibited pathway after BTZ treatment was the E2F target, which is known to play an essential role in cell proliferation and survival in cancer. Negative enrichment was observed in other oncogenic pathways such as MYC targets and oxidative phosphorylation (Fig. 7C). Among the top 50 upregulated genes using the proteasome inhibitor, genes involved in the innate immune signalling, such as *ISG20*, in addition to chemokines, such as *CXCL3* and *CXCL8*. Other genes involved in fatty acid metabolism such as *PALM3*, *OLAH* was a marked upregulation in the *ATF3* and the E3‐ubiquitin ligase *TRIM54*. Furthermore, downregulation of genes from Wnt‐β catenin signalling, such as *WNT7B* and *CTNNA2*, was observed after proteasome inhibition (Fig. 7D). Indeed, our finding indicates that oncogenic and resistance pathways that were associated with high proteasome expression were downregulated when cancer cells are treated with proteasome inhibitors. In previous study, it was found that targeting proteasome or immunoproteasome subunits that caused reduced proteolytic activity was associated with damaged cell function [14]. We conducted experiments using siRNAs in THP1 cells that did affect proteolytic activity in these cells (PSMB6, PSMD4, and ADRM1). Our results demonstrated that a single proteasome subunit has no significant impact on cell viability (Fig. S3). However, complete inhibition of the proteasome has been shown to lead to cell death (Fig. S4).

### 3.7 Correlation between gene expression of the proteasome 20S subunits and ABC transporters

**Fig. 8:**
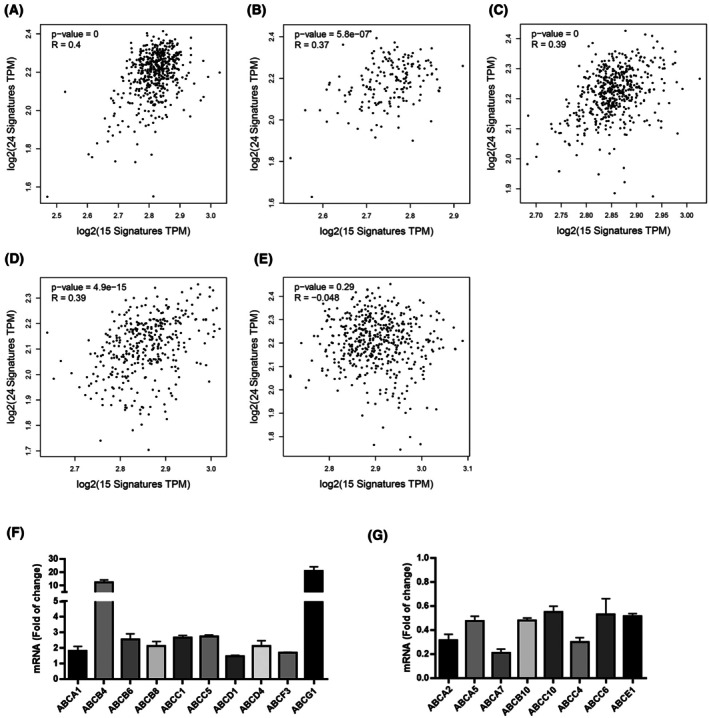
Correlation between proteasome 20S subunits expression and ABC transporters. (A–E) Correlation obtained using the GEPIA2 database between the signature of proteasome (15 genes) and the signature of ABC transporters (24 genes). The results were considered significant if *P* ≤ 0.05 and Pearson ≥ 0.3. (F, G) Transcriptome of ABC transporter in THP‐1 cells after 16 h of exposure to 10 nm of bortezomib (*n* = 3 biological replicates). The results are shown as fold of changes of RPKM in treated cells when compared to control. ANONA one‐way was used for statistical analysis. Only significant results are shown (*P* ≤ 0.05). Results are presented as mean ± SD.

ABC proteins are a family of transporters that participate in the reduction of intracellular concentrations of antitumour medications and therefore participate in the resistance developed against cancer treatment. However, the correlation of proteasome expression and ABC transporters is not fully studied. Among the most well‐studied proteins from this family are *ABCG2* (*BCRP*), *ABCB1* (*MDR1*), and ABCC1 (*MRP1*). In this study we created a signature of 24 transporters of the ABC proteins family (Table S9). We used a TCGA based‐GEPIA2 database to create a correlation between the expression of proteasome subunits and the ABC transporter. A positive significant correlation was observed in KIRC, LAML, LGG, and LIHC (Fig. 8A–D). However, this correlation was not observed in LUAD (Fig. 8E). The effect of proteasome inhibition by bortezomib on the expression of ABC transporters in THP‐1 cells was variable (Fig. 8F,G). The ABCCs subfamily of the efflux transporters is known to be involved in the resistance of conventional and adjacent cancer therapy [50]. Several genes involved in drug resistance, such as *ABCC1* and *ABCC5*, were slightly, however, significantly, upregulated (Fig. 8F), which could indicate that they could participate in the resistance to BTZ. Other ABC transporters such as *ABCC4* and *ABCC6* were downregulated after the treatment of these cells (Fig. 8G). In this cell line, the expression levels of *ABCB1* and *ABCG2* were below the detection threshold.

## 4 Discussion

Proteasome inhibitors have emerged as novel therapeutic strategies in cancer management in the last two decades. These medications are currently the first‐line treatments of hematopoietic malignancies [51, 52]. The success of proteasome inhibitors in solid tumours is still limited and several clinical trials are still ongoing to estimate the effect in combination with other treatments. Therefore, it is essential to understand how proteasome expression is changed in cancer and which solid tumours could benefit from proteasome inhibition. In this study, we used the publicly available TCGA data to filter out tumour entities in which high expression of proteasome subunits is associated with worse patient survival. We have found that the effect of proteasome subunits expression on patient survival is cancer type‐dependent. In LGG, we found a significant association between high transcriptome levels of *PSMA6* and *PSMB5* and better prognosis. This effect is probably dependent on the cancer subtype. In ovarian cancer, and to a lesser degree in thyroid cancer, we found a better survival in patients with high proteasome expression. Indeed, a phase II‐trial for ovarian cancer to establish the benefit of a combination of BTZ with doxorubicin was terminated because the antitumor activity failed [53]. However, other components of the UPS system, such as E3 ligases, were elevated in the epithelial type of ovarian cancer. These tumours could benefit from specific inhibitors of E3‐ligases [54]. A previous analysis of PSMAs in breast cancer has indicated that, except for *PSMA5*, the expression of standard proteasome α‐subunits was associated with worse survival [55]. Another study has pointed out the prognostic value of the β‐subunits of the SP and IP in renal cell kidney cancer [25]. Gene expression of 20S proteasome α‐ and β‐subunits is mainly driven by the transcription factor NFE2L1/Nrf1/TCF11 in a concerted manner [56]. Therefore, we evaluated the effect of 20S proteasome gene expression as a functional unit. We concentrated on cancer types, in which 20S proteasome subunit expression is consistently associated with worse survival. Further studies are planned to evaluate the role of the regulatory particle (19S) subunits in cancer.

Most studies of the proteasome and the ubiquitin proteasome system are conducted in the malignant stages. However, there are indicators that UPS plays an important role in cancer initiation [57]. Proteasomes play a critical role in several oncogenic pathways that contribute to the initiation of tumours, including controlling the abundance of the tumour suppressor p53, NF‐κB, or the oncogenic transcription factors of the β‐catenin pathway. Consequently, higher activity of the proteasome is associated with lower levels of p53, and higher levels of NF‐κB and β‐catenin, which significantly contribute to tumourigenesis [58, 59, 60, 61].

DNA methylation has been found to have a significant impact on the expression of several proteasome subunits, particularly, in LGG and LIHC (Fig. 3). DNA methylation of *PSMA7* detected in the circulating DNA obtained from plasma of LIHC patients could be used as a prognosis marker [62]. It is worth mentioning that proteasome activity has an impact on epigenetic features, including histone acetylation, the chromatin dynamics or stability, and DNA methylation [63]. It is known that DNA methyltransferase 1 is degraded by the proteasome [64]. Alteration in the DNA methylation profile has been recently found to be associated with resistance to BTZ in haematological cancer cells. The combination of DNA methylation inhibitors with BTZ have reduced the cell proliferation and caused the restoration of sensitivity to BTZ in cancer cells and multiple myeloma patients [65, 66]. However, the addition of bortezomib to DAC did not improve the therapeutic outcome in acute myeloid leukaemia patients [67]. Both BTZ and DNA methylation inhibitors are known to target the NF‐κB pathway. Therefore, a synergic effect of DNA methylation inhibitor and BTZ is expected [68]. Our results, validated by DAC treatment, indicate that lower DNA methylation of the proteasome genes is often associated with higher expression of the proteasome subunits (Fig. 3F).

Several variants identified in the TCGA somatic sequencing data were predicted to disrupt proteasome function (Fig. 4). In addition, it is worth mentioning that an additive effect of multiple distinct subunit variants has been demonstrated to result in proteasomal loss of function and different diseases [14]. In this context, it is plausible to conclude that the collective presence of all the core particle variants found in sample TCGA‐DU‐6392 leads to proteasome impairment in the cancer cell, thereby contributing to the observed survival of this patient. Whether this effect occurs at a functional level or during proteasome assembly remains to be elucidated.

The UPS plays an essential role in various cellular functions, including cell cycle, gene transcription, apoptosis, protein quality control, and epigenetic modifications [7, 63, 69]. The induction of proteasome subunits after treatment with PIs is a complex response that can have both prosurvival and cell death outcomes, depending on the cellular context and the extent of proteasome inhibition. Proteasome impairment triggers proteotoxic stress pathways such as the unfolded protein response (UPR), the integrated stress response (ISR), the induction of type‐I interferon signalling, and the activation of the p97/NGLY1/DDI2/NFE2L1 axis. The activation of these pathways results in the induction of various transcription factors such as ATF3, ATF4, and ATF6, which cause transcriptional activation of rescue factors, or could cause metabolic modifications, dysregulated immune signalling, and cell death [3, 70]. The UPR induced by PIs is generally considered a prosurvival mechanism, as it aims to restore cellular homeostasis. However, persistent or excessive induction of the UPR can lead to cell death due to the overwhelming proteotoxic stress [71, 72, 73]. Similarly, the induction of proteasome subunits after proteasome inhibition might initially support cell survival by attempting to compensate for impaired proteasome function, but if proteasome activity remains insufficient, it can trigger apoptotic pathways [74].

The higher expression of proteasome 20S subunits was combined with elevated survival pathways activation such as MYC targets, E2F targets, and DNA repair. In contrast, BTZ leads to a significant reduction in the survival pathways and to the activation of apoptotic pathways. Metabolic pathways such as fatty acid metabolism, adipogenesis, and glycolysis were enriched in the cohorts with high proteasome expression and reduced after treatment with BTZ. In addition, it has been reported that proteasome dysfunction disrupts adipogenesis and affects lipid metabolism [75].

There are controversial results about the interaction between proteasome inhibitors and the ABC‐family of transporters. For example, proteasome inhibition was found to reverse the resistance of vincristine‐resistant human gastric cancer cell line by the inhibition of multidrug resistance MDR1 encoded by *ABCB1* [76]. Further studies have reported the capacity of BTZ to reduce the expression of these resistance markers, such as MDR1 and MRP1 [77], MDR1 via the inhibition of NF‐κB pathway [78]. In our study, we have found that in THP‐1 cells, BTZ did induce the expression of MRP1, which could contribute to BTZ resistance. However, we found a reduction in the expression of other efflux transporters such as MRP4 (ABCC4). These results suggest that BTZ could reduce the resistance to drugs that are substrates for these transporters such as 5‐fluorouracil.

Our findings mainly indicate the importance of studying the proteasome as an enzymatic functional unit rather than separated subunits. In addition, our study pointed out types of cancer that could be candidates for PI treatment.

## 5 Conclusion

In general, our study pointed out that, albeit the effect that is observed for specific proteasome subunits on cellular function, the main role of proteasome is better evaluated when the proteasome is studied as an enzyme unit. In this comprehensive study, we defined cancer types in which proteasome subunits expression is associated with worse survival. These types of cancer are candidates to develop therapeutic strategies that aim to inhibit the proteasomal activity or induce proteotoxic stress. We also detected DNA methylation sites that are associated with reduced expression. In addition to the well‐defined pathways that could be affected by proteasome expression, we explored the novel correlation between proteasome subunit expression, the expression of ABC transporters, and stem cells markers in various types of cancer.

## Conflict of interest

The authors declare no conflict of interest.

## Author contributions

EK and R Al‐Abdulla contributed to article design (data and experimental design); R Al‐Abdulla, SV, and R Al‐Ali contributed to data processing; MW, SV, and R Al‐Abdulla contributed to mutations data; R Al‐Abdulla, SV, and EK contributed to article preparation; R Al‐Abdulla and MR contributed to RNA‐seq experiment; R Al‐Abdulla and R Al‐Ali contributed to RNA‐seq analysis; R Al‐Abdulla, EK, and SV contributed to statistical analysis.

## Supporting information


**Fig. S1.** Electrostatic potential and surface hydrophobicity of the variants discovered in TCGA‐DU‐6392, visualized in ChimeraX.
**Fig. S2.** Graphical summary from the IPA core analysis.
**Fig. S3.** siRNA treatment of THP‐1 cells.
**Fig. S4.** Cell viability assay after Bortezomib treatment in THP‐1 cells.
**Table S1.** TCGA abbreviations and number of samples used in the survival study.
**Table S2.** Proteins encoded by the proteasome subunits genes.
**Table S3.** Primer sequences for qPCR.
**Table S4.** Comprehensive variant evaluation and selection process.
**Table S5.** Correlation between overall and proteasome mutations and Kaplan–Meier survival.
**Table S6.** Identification of potentially disruptive variants in proteasome‐associated genes.
**Table S7.** Differential expression factors of gene of interest by mean values.
**Table S8.** Number of classified patients out of the whole cancer specific patient number.
**Table S9.** 24 transporters of the ABC proteins family.
**Data S1.** Data from TCGA and data from Cox‐regression analysis.

## Data Availability

The FastQ files of the RNA‐seq experiment is shared in GEO under PRJNA1032858.
