# Supplementary material for: Expression and DNA methylation of 20S proteasome subunits as prognostic and resistance markers in cancer
Source: Mol Oncol. 2025 Aug 27;19(12):3729–49. doi: 10.1002/1878-0261.70038 (PMC12688171; doi:10.1002/1878-0261.70038)

Supplementary material

Expression and DNA methylation of 20S proteasome subunits as prognostic and resistance markers in cancer

Ruba Al-Abdulla[1], Simone Venz[1], Ruslan Al-Ali[2], Martin Wendlandt[1], Mandy Radefeldt[2], Elke Krüger[1]

1. Institute of Medical Biochemistry and Molecular Biology, University Medicine of Greifswald, Greifswald, Germany. ruba.alabdulla@med.uni-greifswald.de (R.AA), Simone.Venz@med.uni-greifswald.de (S.V), Martin.Wendlandt@med.uni-greifswald.de (M.W), Elke.Krueger@med.uni-greifswald.de (E.K)
2. Centogene, Rostock, Germany. Ruslan.Al-Ali@centogene.com (R.A), Mandy.Radefeldt@centogene.com (M.R).

Corresponding Author: Ruba Al-Abdulla

Ruba.alabdulla@med.uni-greifswald.de

Tel. +49 03834/865404

Elke Krüger,

Elke.Krueger@med.uni-greifswald.de

Tel. +49 03834/865400

Supplementary Table of Content:

**Supplementary Table S1**	**TCGA abbreviations and number of samples used in the survival study.**
**Supplementary Table S2**	Proteins encoded by the proteasome subunits genes.
**Supplementary Table S3**	Primer sequences for qPCR.
**Supplementary Table S4**	given as Excel-file<br>Comprehensive variant evaluation and selection process
**Supplementary Table S5**	given as Excel-file<br>Correlation between overall and proteasome mutations and Kaplan-Meier survival.
**Supplementary Table S6**	Identification of potentially disruptive variants in proteasome-associated genes.
**Supplementary Table S7**	Differential expression factors of gene of interest by mean values.
**Supplementary Table S8**	Number of classified patients out of the whole cancer specific patient number.
**Supplementary Table S9**	24 transporters of the ABC proteins family.
**Supplementary Figure S1**	Electrostatic potential and surface hydrophobicity of the variants discovered in TCGA-DU-6392, visualized in ChimeraX.
**Supplementary Figure S2**	Graphical summary from the IPA core analysis.
**Supplementary Figure S3**	siRNA treatment of THP-1 cells.
**Supplementary Figure S4**	Cell viability assay after Bortezomib treatment in THP-1 cells.
**Supplementary Data S-d1**	Data from TCGA and dato from Cox-regression analysis.

Supplementary Table S1:

TCGA abbreviations and number of samples used in the survival study.

Abbreviation	Cancer type	Nr. of Patients
LAML	Acute Myeloid Leukemia	151
LGG	Low Grade Glioma	511
KIRC	Kidney Renal Cell Carcinoma	538
LIHC	Liver Hepatocellular Carcinoma	371
LUAD	Lung Adenocarcinoma	533
OV	Ovarian Serous Cystadenocarcinoma	374
THYM	Thymoma	119
THCA	Thyroid Carcinoma	502
ESCA	Esophageal Carcinoma	161
HNSC	Head and Neck Squamous Cell Carcinoma	500
KIRP	Kidney Renal papillary Cell Carcinoma	288
LUSC	Lung Squamous Cell Carcinoma	502
PAAD	Pancreatic Adenocarcinoma	177
PRAD	Prostate Adenocarcinoma	498
SARC	Sarcoma	259
STAD	Stomach Adenocarcinoma	375
TGCT	Testicular Germ Cell Tumors	150
UCEC	Uterine Corpus Endometrial Carcinoma	551
BLCA	Bladder Urothelial Carcinoma	414
GBM	Glioblastoma Multiforme	156
BRCA	Breast Invasive Carcinoma	1102
CESC	Cervical Squamous Cell Carcinoma and Endocervical Adenocarcinoma	304
COAD	Colon Adenocarcinoma	478
READ	Rectum Adenocarcinoma	304

Supplementary Table S2:

Proteins encoded by the proteasome subunits genes.

Proteasome alpha subunits	Proteasome beta subunits	ATPase – dependent regulatory particles (RPTs)	ATPase independent regulatory genes	Other Regulators
PSMA1 (α6 subunit)	*PSMB1* (β6 subunit)	*PSMC1* (Rpt2)	*PSMD1* (Rpn2)	*PSME1* (PA28α)
PSMA2 (α2 subunit)	*PSMB2* (β4 subunit)	*PSMC2* (Rpt1)	*PSMD2* (Rpn1)	*PSME2* (PA28β)
PSMA3 (α7 subunit)	*PSMB3* (β3 subunit)	*PSMC3* (Rpt5)	*PSMD3* (Rpn3)	*PSME3* (PA28γ)
PSMA4 (α3 subunit)	*PSMB4* (β7 subunit)	*PSMC4* (Rpt3)	*PSMD4* (Rpn10)	*PSME4* (PA200)
PSMA5(α5 subunit)	*PSMB5* (β5 subunit)	*PSMC5* (Rpt6)	*PSMD6* (Rpn7)	*PSMF1* (PI31)
PSMA6 (α1 subunit)	*PSMB6* (β1 subunit)	*PSMC6* (Rpt4)	*PSMD7* (Rpn8)	
PSMA7 (α4 subunit)	*PSMB7* (β2 subunit)		*PSMD8* (Rpn12)	
PSMA8 (α7-like protein)	*PSMB8* (β5i subunit)		*PSMD11* (Rpn6)	
	PSMB9 (β1i subunit)		*PSMD12* (Rpn5)	
	PSMB10 (β2i, MECL1 subunit)		*PSMD13* (Rpn9)	
	PSMB11 (β5t subunit)		*PSMD14* (Rpn11)	
			ADRM1 (Rpn13)	
			SHFM1 (Rpn15)	

Supplementary Table S3:

Primer sequences for qPCR.

Name	Sequence 5'-->3'	Name	Sequence 5'-->3'
**PSMA1-F**	TTATGCGTCAGGAGTGTTTGG	**PSMB1-F**	GGTGGACTTGATGAAGAAGGAAAG
**PSMA1-R**	GTTGGTATCTGGGTCTTGCTTC	**PSMB1-R**	GTAGCATGGCACTTGCTGAG
**PSMA2-F**	GCGAAGTGTACACAAAGTAGAACC	**PSMB2-F**	TGGAGGAGCTCCAGAAACG
**PSMA2-R**	CTGTGCACAAGCACTCTGTAATC	**PSMB2-R**	CATGATGTTAGGAGCCCTGTTTG
**PSMA3-F**	GCAAAGATGGTGTTGTCTTTGG	**PSMB3-F**	TGACGTCCAGACAGTTGCC
**PSMA3-R**	AAACCTGCTACTGCCATTCC	**PSMB3-R**	CCACCATGCTCATGAGGGTATAAG
**PSMA4-F**	GCTGTACATTGGCTGGGATAAG	**PSMB4-F**	GAGCTATTCATTCATGGCTGACC
**PSMA4-R**	ACAGCTGCAGCGCTATTATTTC	**PSMB4-R**	CGAGGAAGCTCTCTCCATCAG
**PSMA5-F**	TTGGGATCCAGACATCAGAGG	**PSMB5-F**	CTCCAAACTGCTTGCCAAC
**PSMA5-R**	GGCACAACCTATGTGAGCATC	**PSMB5-R**	GGTTCCCTTCACTGTCCAC
**PSMA6-F**	ACATGTCCCGTGGTTCCAG	**PSMB6-F**	ACAACCACTGGGTCCTACATC
**PSMA6-R**	TGTAAGGCCACCCTGGTTAATAG	**PSMB6-R**	ATCAGCTACTGCCTGGGTATC
**PSMA7-F**	CATGCCTGGAAGGCCAATG	**PSMB7-F**	CATGACAACCCAGCTCATTTCTTC
**PSMA7-R**	TGCCTTGATCACCAGCTTAATG	**PSMB7-R**	CATCTGCTTCAGCATCCGATTG

Supplementary Table S6:

Identification of potentially disruptive variants in proteasome-associated genes.
extraction, filtering, and manual classification of TCGA somatic sequencing data

Cancer type	TCGA Tumor Sample	Genes	HGVS	ACMG Class
KIRC	TCGA-B8-5164	POMP	c.162+1G>T	4
	TCGA-BP-4964	PSMA3	c.396dup p.(Gly133Trpfs*13)	4
	TCGA-B0-4823	PSMA4	c.581delinsGCAAAG p.(Ile194Serfs*6)	4
	TCGA-B0-4811	PSMA6	c.77-1G>C	4
	TCGA-B0-5088	PSMB4	c.374_375insT p.(His126Thrfs*4)	4
LAML	-	-	-	-
LGG	TCGA-P5-A77X	PSMA2	c.226T>C p.(Tyr76His)	3
	TCGA-DU-6392	PSMB4	c.257G>A p.(Arg86His)	3
LIHC	TCGA-G3-A3CK	PSMA4	c.356A>T p.(Gln119Leu)	3
	TCGA-ED-A7PZ	PSMA7	c.527A>G p.(Tyr176Cys)	3
	TCGA-2Y-A9H0	PSMB2	c.317G>A p.(Gly106Asp)	3
	TCGA-WQ-A9G7	PSMB3	c.296+2T>C	3
LUAD	TCGA-MN-A4N5	PSMA1	c.238G>T p.(Asp80Tyr)	3
	TCGA-05-4427	PSMA3	c.524C>T p.(Thr175Met)	3
	TCGA-50-6590	PSMA6	c.245G>C p.(Gly82Ala)	3
	TCGA-55-8203	PSMA7	c.348+1G>A	4
	TCGA-78-8660		c.389C>T p.(Ser130Phe)	3

Supplementary Table S7:

Differential expression factors of gene of interest by mean values.
Fold change factor was calculated by the gene expression data of all patients fitting to cancer type. The significance is given by the determined p-value.

**Hugo Symbol**	**KIRC factor**	KIRC pvalue	**LAML factor**	LAML pvalue	**LGG factor**	LGG pvalue	**LIHC factor**	LIHC pvalue	**LUAD factor**	LUAD pvalue
PSMA1	**1.29**	2.16E-30	**1.19**	7.53E-09	**1.19**	1.33E-25	**1.31**	2.83E-21	**1.43**	1.25E-32
PSMA2	**1.32**	3.38E-22	**1.37**	1.77E-15	**1.32**	1.67E-42	**1.35**	6.49E-16	**1.59**	1.94E-41
PSMA3	**1.45**	2.33E-36	**1.32**	3.55E-15	**1.31**	3.98E-39	**1.48**	1.11E-30	**1.64**	1.58E-32
PSMA4	**1.46**	2.39E-53	**1.25**	1.25E-09	**1.30**	1.37E-31	**1.56**	9.72E-38	**1.54**	3.25E-39
PSMA5	**1.51**	1.05E-47	**1.42**	9.44E-22	**1.34**	7.87E-34	**1.61**	7.66E-26	**1.68**	2.05E-48
PSMA6	**1.32**	5.50E-10	**1.28**	6.48E-12	**1.28**	9.80E-35	**1.52**	6.07E-35	**1.82**	1.06E-15
PSMA7	**1.49**	2.70E-18	**1.46**	3.14E-19	**1.39**	1.35E-50	**1.68**	2.19E-35	**1.62**	6.49E-33
PSMB1	**1.49**	5.51E-47	**1.32**	1.03E-24	**1.36**	1.93E-36	**1.94**	9.29E-36	**1.66**	6.69E-48
PSMB2	**1.32**	2.35E-34	**1.10**	3.75E-03	**1.20**	5.97E-18	**1.49**	4.61E-25	**1.42**	3.48E-36
PSMB3	**1.97**	8.75E-42	**1.48**	1.85E-15	**1.56**	5.99E-45	**2.13**	2.95E-32	**1.79**	4.94E-32
PSMB4	**1.63**	4.25E-53	**1.36**	2.30E-16	**1.37**	7.69E-48	**1.68**	4.37E-27	**1.58**	1.93E-33
PSMB5	**1.35**	1.03E-26	**1.42**	3.90E-14	**1.23**	1.52E-21	**1.39**	1.99E-24	**1.67**	3.09E-32
PSMB6	**1.63**	5.09E-44	**1.48**	1.58E-21	**1.40**	4.37E-43	**1.85**	1.80E-27	**1.64**	2.75E-37
PSMB7	**1.49**	4.11E-41	**1.31**	5.56E-17	**1.31**	3.96E-29	**1.84**	2.33E-35	**1.63**	1.14E-44
POMP	**1.46**	1.91E-54	**1.51**	1.34E-15	**1.30**	2.37E-31	**1.75**	1.80E-37	**1.68**	5.68E-24

Supplementary Table S8:

Number of classified patients out of the whole cancer specific patient number. To pass the IPA criteria for the pathway analysis a cut off factor of 1.5 fold was used to diminish the number of different genes within the cancer types.

**Disease**	**No. of expressed genes (TCGA)**	**No. of differentially expressed genes factor 1.5**
**KIRC**	17637	4925
**LAML**	17729	2119
**LGG**	17607	3790
**LIHC**	17499	5834
**LUAD**	17582	4534

Supplementary Table S9:

24 transporters of the ABC proteins family. These proteins were used to create a correlation between the expression of proteasome subunits and ABC transporter.

**Gene Names (primary)**	**Entry**	**Entry Name (HUMAN)**	**Protein names**
ABCA1	O95477	ABCA1	Phospholipid-transporting ATPase ABCA1
ABCA2	Q9BZC7	ABCA2	ATP-binding cassette sub-family A member 2
ABCA3	Q99758	ABCA3	Phospholipid-transporting ATPase ABCA3
ABCA5	Q8WWZ7	ABCA5	Cholesterol transporter ABCA5
ABCA7	Q8IZY2	ABCA7	Phospholipid-transporting ATPase ABCA7
ABCB10	Q9NRK6	ABCBA	ATP-binding cassette sub-family B member 10, mitochondrial
ABCB4	P21439	MDR3	Phosphatidylcholine translocator ABCB4
ABCB6	Q9NP58	ABCB6	ATP-binding cassette sub-family B member 6
ABCB7	O75027	ABCB7	Iron-sulfur clusters transporter ABCB7, mitochondrial
ABCB8	Q9NUT2	MITOS	Mitochondrial potassium channel ATP-binding subunit
ABCB9	Q9NP78	ABCB9	ABC-type oligopeptide transporter ABCB9
ABCC1	P33527	MRP1	Multidrug resistance-associated protein 1
ABCC10	Q5T3U5	MRP7	ATP-binding cassette sub-family C member 10
ABCC4	O15439	MRP4	ATP-binding cassette sub-family C member 4
ABCC5	O15440	MRP5	ATP-binding cassette sub-family C member 5
ABCC6	O95255	MRP6	ATP-binding cassette sub-family C member 6
ABCD1	P33897	ABCD1	ATP-binding cassette sub-family D member 1
ABCD3	P28288	ABCD3	ATP-binding cassette sub-family D member 3
ABCD4	O14678	ABCD4	Lysosomal cobalamin transporter ABCD4
ABCE1	P61221	ABCE1	ATP-binding cassette sub-family E member 1
ABCF1	Q8NE71	ABCF1	ATP-binding cassette sub-family F member 1
ABCF2	Q9UG63	ABCF2	ATP-binding cassette sub-family F member 2
ABCF3	Q9NUQ8	ABCF3	ATP-binding cassette sub-family F member 3
ABCG1	P45844	ABCG1	ATP-binding cassette sub-family G member 1

Supplementary Figure S1:

Electrostatic potential and surface hydrophobicity of the variants discovered in TCGA-DU-6392, visualized in ChimeraX. (A-F) Comparison of the electrostatic potential (top panel) and hydrophobicity (bottom panel) between the reference structure of each subunit (left) and the corresponding folded variant structure (right).

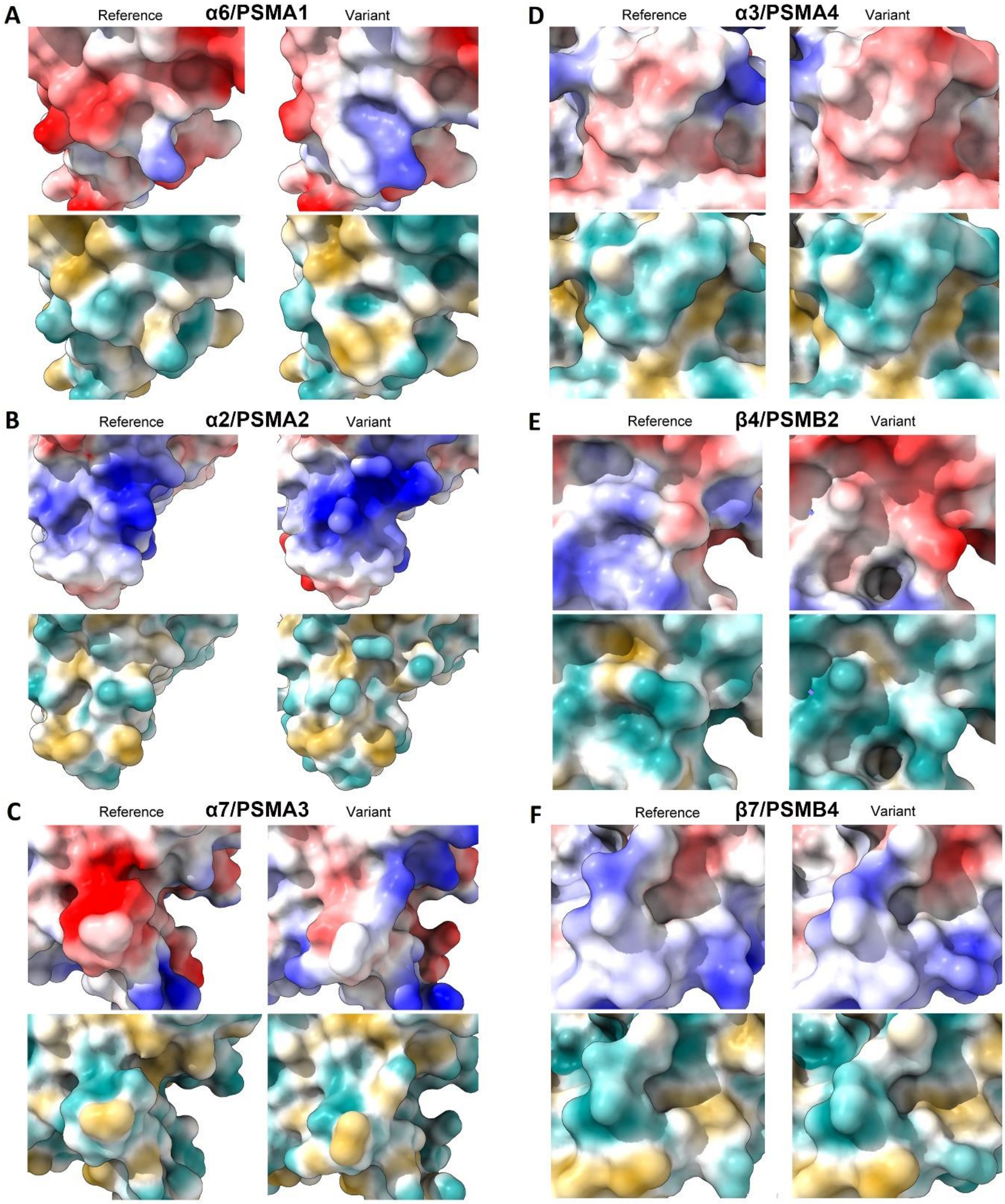

Supplementary Figure S2:

Graphical summary from the IPA core analysis.
Summary of key results from the IPA core analysis for each type of cancer presented in a network. Predicted activation were coloured with orange symbols and lines. Predicted inactivation were coloured with blue symbols and lines. Marks in grey fit to interaction behavior.

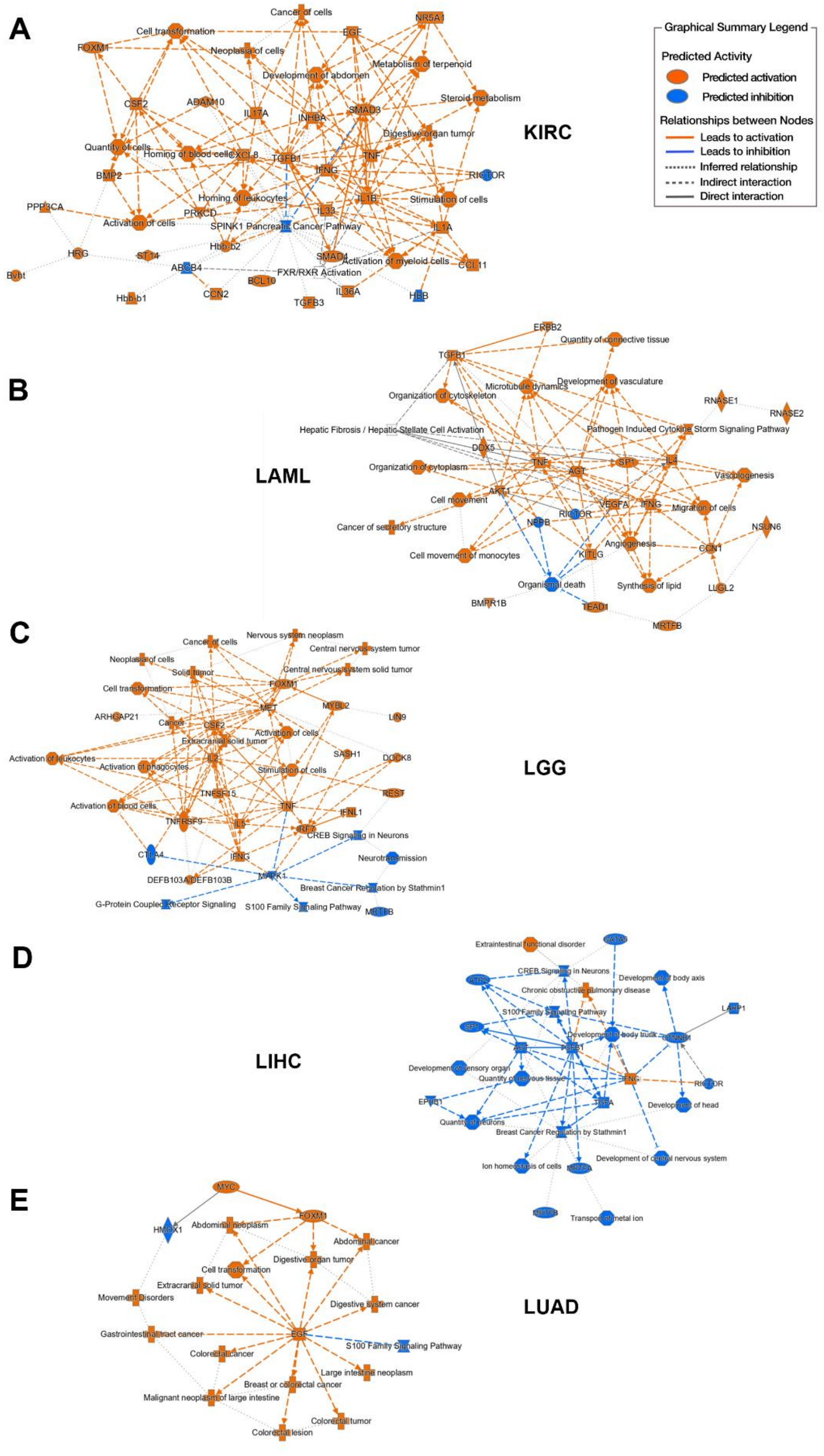

Supplementary Figure S3:

siRNA treatment of THP-1 cells.
(A) Different immunoblots were given for different proteasomal subunits after transfection with corresponding siRNA
(B) cell viability assay using WST-reagent

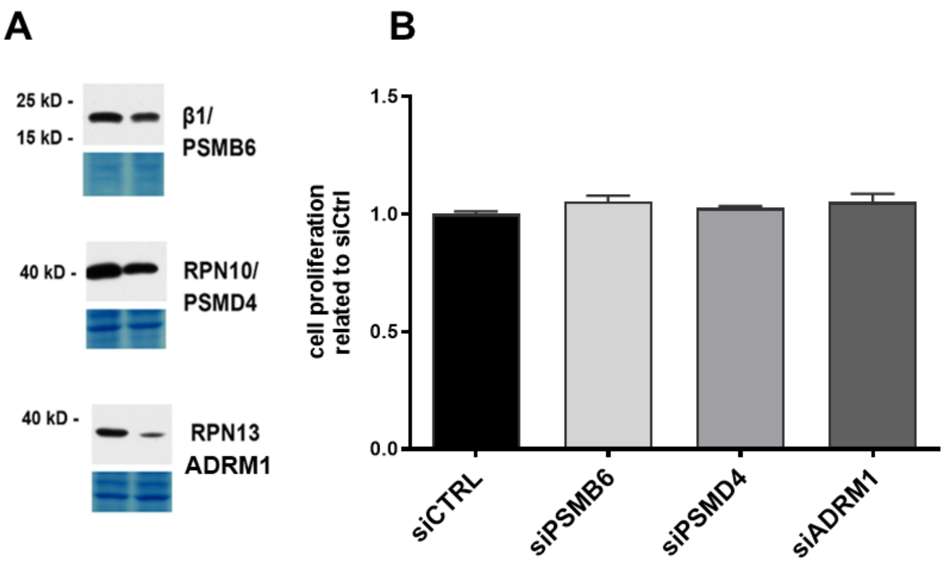

Supplementary Figure S4:

Cell viability assay after Bortezomib treatment in THP-1 cells.
Incubation of THP-1 cells with increased concentrations of Bortezomib (BTZ) for 72 hrs. Cell viability assay was performed using WST-reagent.

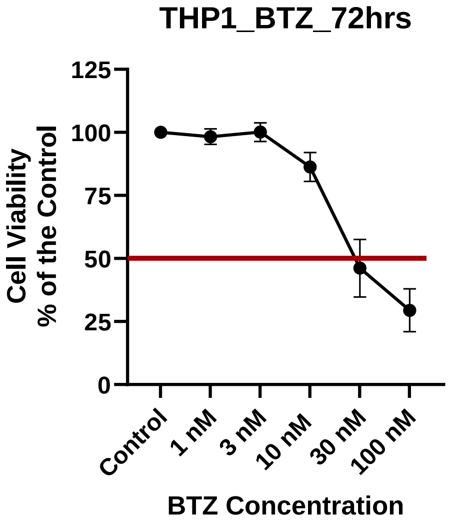

Supplementary data S-d1:

All data comes from TCGA PanAtlas 2018 (cBioPortal)

Code comes from tutorial:

https://rviews.rstudio.com/2017/09/25/survival-analysis-with-r/

The code in R for LAML:

```
library(survival)

library(ranger)

library(ggplot2)

library(dplyr)

library(ggfortify)

library(reshape2)

#------------

rna=read.csv("laml_tcga_pan_can_atlas_2018/data_mrna_seq_v2_rsem.txt",sep="\t")

row.names(rna)=make.names(rna$Hugo_Symbol,unique = T)

LAML_1=rna[c('PSMA1','PSMA2','PSMA3','PSMA4','PSMA5','PSMA6','PSMA7',"PSMB1","PSMB2","PSMB3","PSMB4","PSMB5","PSMB6","PSMB7"), 3:ncol(rna) ]

clinical=read.csv("laml_tcga_pan_can_atlas_2018/data_clinical_patient.txt",sep="\t",row.names = 1)

clinical=clinical[5:nrow(clinical) ,29:35]

colnames(clinical)=
c("Overall.Survival.Status","Overall.Survival..Months.","Disease.specific.Survival.status","Months.of.disease.specific.survival")

x=colsplit(row.names(clinical),pattern = "-",names = c("t","A","number","x"))[,2:3]

x$con=paste0(x[,1],"-",x[,2])

row.names(clinical)=x$con

x=colsplit(colnames(LAML_1),pattern = "[.]",names = c("t","A","number","x"))[,2:3]

x$con=paste0(x[,1],"-",x[,2])

colnames(LAML_1)=x$con

LAML=as.data.frame(t(LAML_1))

LAML$sur=as.numeric(clinical[row.names(LAML),2])

clinical$Status= factor(clinical$Overall.Survival.Status,labels = c(0,1))

LAML$status=clinical[row.names(LAML),'Status']

LAML=as.data.frame(LAML)

LAML$id=row.names(LAML)

LAML=LAML[,c(17,15,16,1:14)]

LAML$status=as.numeric(as.character(LAML$status))

formula <- Surv(sur, status) ~ PSMA1 + PSMA2 + PSMA3+ PSMA4 + PSMA5+ PSMA6 + PSMA7
+PSMB1 +PSMB2 +PSMB3 +PSMB4 +PSMB5 +PSMB6 +PSMB7

cox_model <- coxph(formula, data = LAML)
```

summary(cox_model)

cox_fit <- survfit(cox_model)

autoplot(cox_fit)

aa_fit <-aareg(Surv(sur, status) ~ PSMA1 + PSMA2 + PSMA3+ PSMA4 + PSMA5+ PSMA6 + PSMA7 +PSMB1 +PSMB2 +PSMB3 +PSMB4 +PSMB5 +PSMB6 +PSMB7,

data = LAML)

aa_fit

autoplot(aa_fit)

LAML

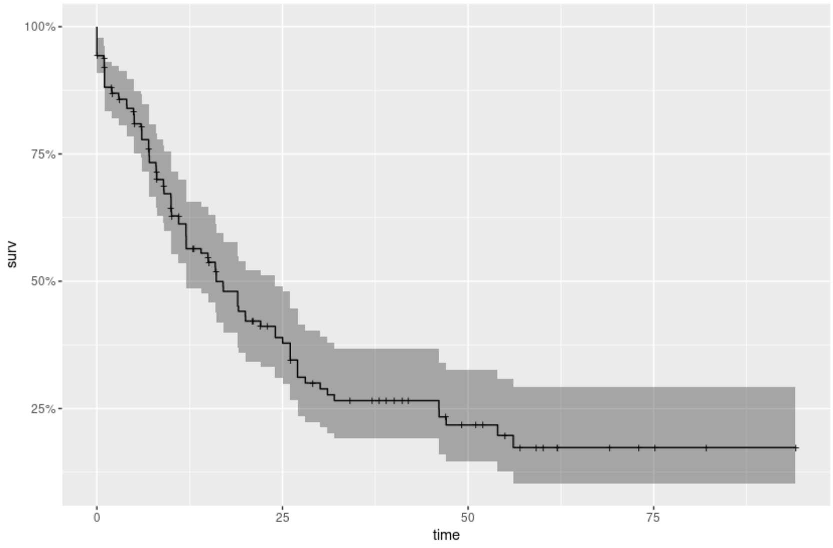

Call:

coxph(formula = formula, data = LAML)

```
  n= 161, number of events= 103
   (12 observations deleted due to missingness)

           coef  exp(coef)   se(coef)      z Pr(>|z|)
PSMA1  3.194e-04  1.000e+00  3.459e-04  0.924  0.35572
PSMA2 -1.057e-03  9.989e-01  3.848e-04 -2.747  0.00601 **
PSMA3  1.025e-03  1.001e+00  9.628e-04  1.065  0.28703
PSMA4 -6.473e-05  9.999e-01  3.663e-04 -0.177  0.85974
PSMA5  5.161e-05  1.000e+00  5.674e-04  0.091  0.92753
PSMA6  2.453e-04  1.000e+00  2.272e-04  1.080  0.28031
PSMA7  4.812e-04  1.000e+00  1.846e-04  2.607  0.00914 **
PSMB1  2.094e-04  1.000e+00  5.309e-04  0.394  0.69333
PSMB2  9.334e-06  1.000e+00  3.318e-04  0.028  0.97756
PSMB3  1.107e-05  1.000e+00  6.283e-04  0.018  0.98594
PSMB4  3.998e-04  1.000e+00  2.715e-04  1.472  0.14090
PSMB5  1.639e-04  1.000e+00  5.485e-04  0.299  0.76511
PSMB6 -1.030e-03  9.990e-01  7.965e-04 -1.293  0.19592
PSMB7 -1.252e-03  9.987e-01  5.227e-04 -2.396  0.01659 *
---
Signif. codes:  0 ‘***’ 0.001 ‘**’ 0.01 ‘*’ 0.05 ‘.’ 0.1 ‘ ’ 1
```

	exp(coef)	exp(-coef)	lower .95	upper .95
PSMA1	1.0003	0.9997	0.9996	1.0010
PSMA2	0.9989	1.0011	0.9982	0.9997
PSMA3	1.0010	0.9990	0.9991	1.0029
PSMA4	0.9999	1.0001	0.9992	1.0007
PSMA5	1.0001	0.9999	0.9989	1.0012
PSMA6	1.0002	0.9998	0.9998	1.0007
PSMA7	1.0005	0.9995	1.0001	1.0008
PSMB1	1.0002	0.9998	0.9992	1.0013
PSMB2	1.0000	1.0000	0.9994	1.0007
PSMB3	1.0000	1.0000	0.9988	1.0012
PSMB4	1.0004	0.9996	0.9999	1.0009
PSMB5	1.0002	0.9998	0.9991	1.0012
PSMB6	0.9990	1.0010	0.9974	1.0005
PSMB7	0.9987	1.0013	0.9977	0.9998

Concordance= 0.636 (se = 0.03)
Likelihood ratio test= 30 on 14 df, p=0.008
Wald test = 31.96 on 14 df, p=0.004
Score (logrank) test = 32.72 on 14 df, p=0.003

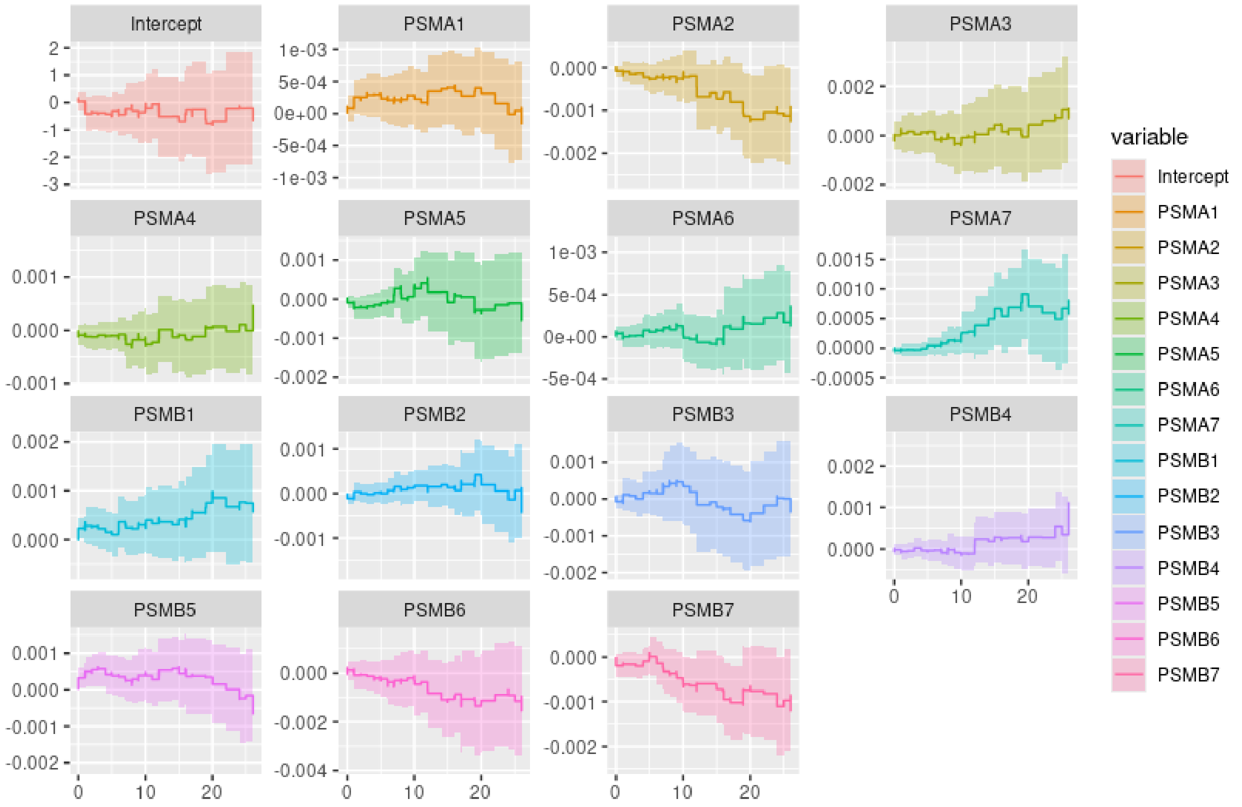

LIHC

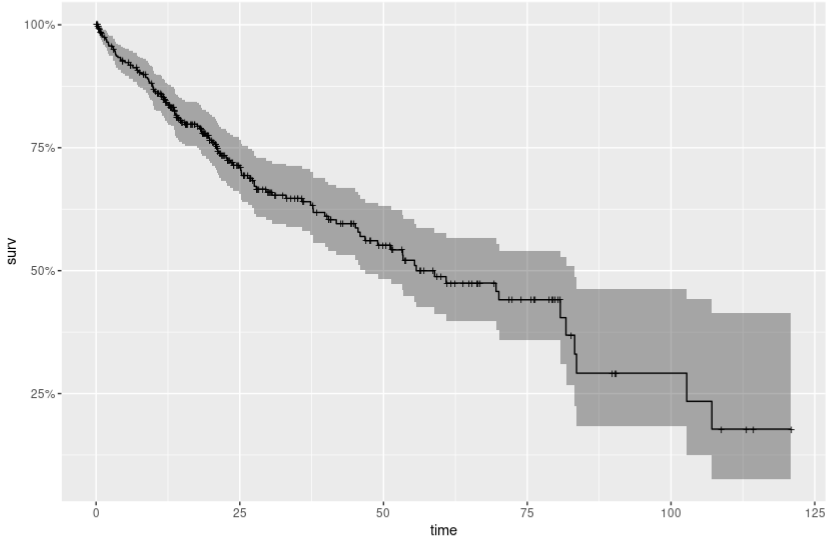

coxph(formula = formula, data = LAML)

n= 365, number of events= 129
(1 observation deleted due to missingness)

	coef	exp(coef)	se(coef)	z	Pr(>|z|)	
PSMA1	3.626e-04	1.000e+00	1.635e-04	2.218	0.0265	*
PSMA2	-1.149e-04	9.999e-01	1.023e-04	-1.124	0.2611	
PSMA3	2.806e-04	1.000e+00	1.720e-04	1.631	0.1028	
PSMA4	-2.631e-04	9.997e-01	1.406e-04	-1.872	0.0612	.
PSMA5	3.059e-05	1.000e+00	9.003e-05	0.340	0.7340	
PSMA6	-1.797e-04	9.998e-01	1.465e-04	-1.227	0.2199	
PSMA7	3.759e-05	1.000e+00	6.423e-05	0.585	0.5584	
PSMB1	-2.508e-05	1.000e+00	6.715e-05	-0.374	0.7087	
PSMB2	2.536e-04	1.000e+00	9.872e-05	2.569	0.0102	*
PSMB3	7.416e-05	1.000e+00	5.784e-05	1.282	0.1998	
PSMB4	-4.549e-06	1.000e+00	2.635e-05	-0.173	0.8629	
PSMB5	1.745e-04	1.000e+00	1.077e-04	1.620	0.1053	
PSMB6	4.675e-06	1.000e+00	9.591e-05	0.049	0.9611	
PSMB7	-1.744e-04	9.998e-01	9.269e-05	-1.881	0.0599	.

Signif. codes: 0 ‘***’ 0.001 ‘**’ 0.01 ‘*’ 0.05 ‘.’ 0.1 ‘ ’ 1

	exp(coef)	exp(-coef)	lower .95	upper .95
PSMA1	1.0004	0.9996	1.0000	1.001
PSMA2	0.9999	1.0001	0.9997	1.000
PSMA3	1.0003	0.9997	0.9999	1.001
PSMA4	0.9997	1.0003	0.9995	1.000
PSMA5	1.0000	1.0000	0.9999	1.000
PSMA6	0.9998	1.0002	0.9995	1.000
PSMA7	1.0000	1.0000	0.9999	1.000
PSMB1	1.0000	1.0000	0.9998	1.000
PSMB2	1.0003	0.9997	1.0001	1.000
PSMB3	1.0001	0.9999	1.0000	1.000
PSMB4	1.0000	1.0000	0.9999	1.000
PSMB5	1.0002	0.9998	1.0000	1.000
PSMB6	1.0000	1.0000	0.9998	1.000
PSMB7	0.9998	1.0002	0.9996	1.000

Concordance= 0.656 (se = 0.025)
Likelihood ratio test= 30.81 on 14 df,   p=0.006

Wald test   = 32.56 on 14 df,   p=0.003
Score (logrank) test = 32.4 on 14 df,   p=0.004

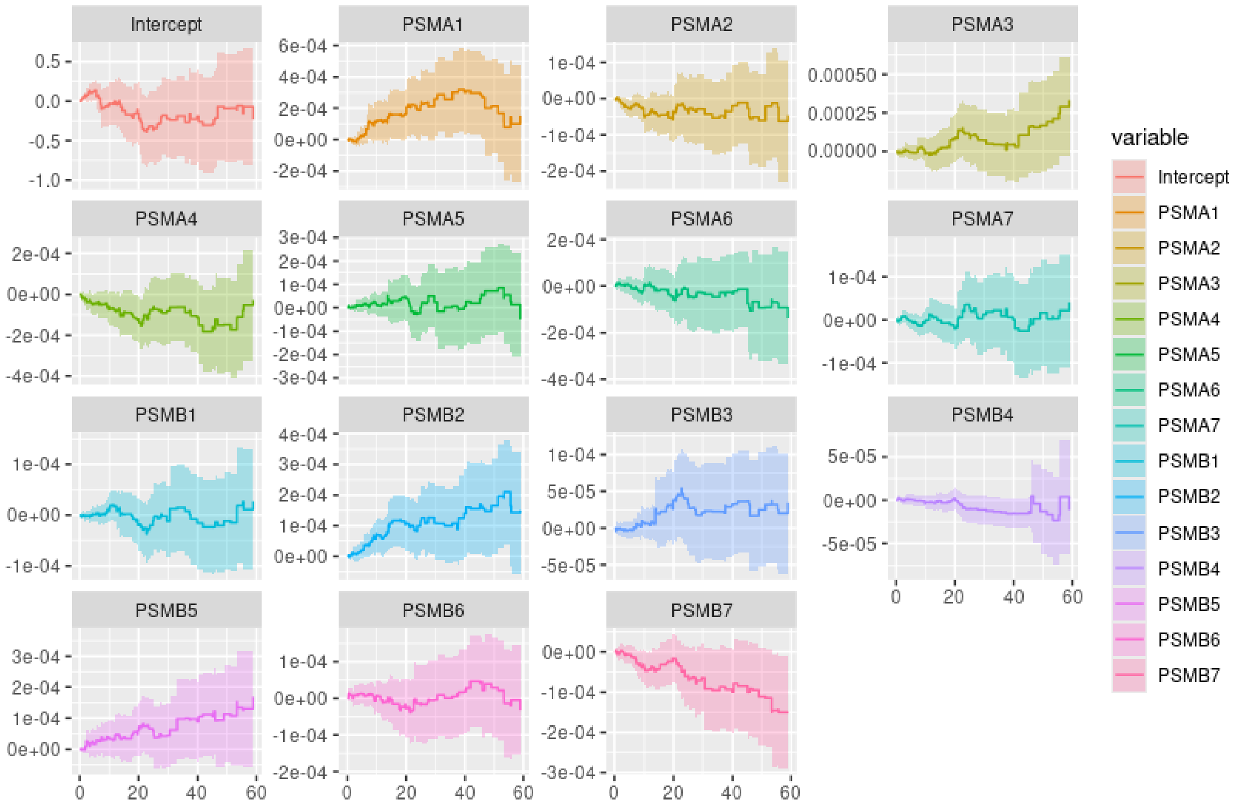

KIRC

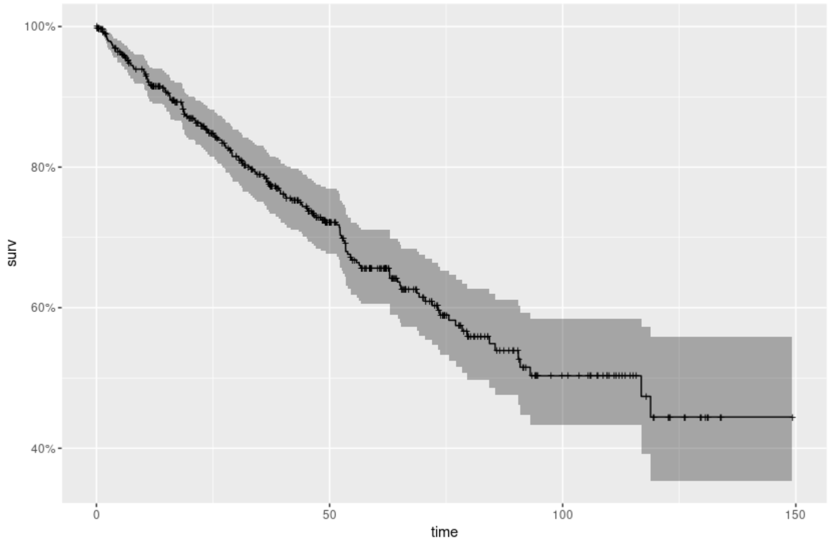

Call:
coxph(formula = formula, data = LAML)
n= 510, number of events= 168

	coef	exp(coef)	se(coef)	z	Pr(>|z|)	
PSMA1	7.003e-04	1.001e+00	2.410e-04	2.905	0.00367	**
PSMA2	-5.301e-04	9.995e-01	1.743e-04	-3.041	0.00236	**
PSMA3	-1.962e-04	9.998e-01	3.155e-04	-0.622	0.53408	
PSMA4	6.290e-04	1.001e+00	2.476e-04	2.540	0.01107	*
PSMA5	-4.617e-04	9.995e-01	3.494e-04	-1.321	0.18644	

PSMA6 -2.267e-04 9.998e-01 1.982e-04 -1.144 0.25267
PSMA7 -2.013e-05 1.000e+00 1.556e-04 -0.129 0.89707
PSMB1 -2.530e-05 1.000e+00 1.399e-04 -0.181 0.85645
PSMB2 6.461e-04 1.001e+00 3.016e-04 2.143 0.03215 *
PSMB3 -2.586e-04 9.997e-01 1.735e-04 -1.491 0.13601
PSMB4 1.810e-04 1.000e+00 7.402e-05 2.445 0.01448 *
PSMB5 1.366e-04 1.000e+00 1.585e-04 0.862 0.38866
PSMB6 -1.221e-04 9.999e-01 2.630e-04 -0.464 0.64243
PSMB7 2.004e-04 1.000e+00 1.636e-04 1.225 0.22067

Signif. codes: 0 ‘***’ 0.001 ‘**’ 0.01 ‘*’ 0.05 ‘.’ 0.1 ‘ ’ 1

	exp(coef)	exp(-coef)	lower .95	upper .95
PSMA1	1.0007	0.9993	1.0002	1.0012
PSMA2	0.9995	1.0005	0.9991	0.9998
PSMA3	0.9998	1.0002	0.9992	1.0004
PSMA4	1.0006	0.9994	1.0001	1.0011
PSMA5	0.9995	1.0005	0.9989	1.0002
PSMA6	0.9998	1.0002	0.9994	1.0002
PSMA7	1.0000	1.0000	0.9997	1.0003
PSMB1	1.0000	1.0000	0.9997	1.0002
PSMB2	1.0006	0.9994	1.0001	1.0012
PSMB3	0.9997	1.0003	0.9994	1.0001
PSMB4	1.0002	0.9998	1.0000	1.0003
PSMB5	1.0001	0.9999	0.9998	1.0004
PSMB6	0.9999	1.0001	0.9994	1.0004
PSMB7	1.0002	0.9998	0.9999	1.0005

Concordance= 0.667 (se = 0.022)
Likelihood ratio test= 63.7 on 14 df,   p=3e-08
Wald test   = 74.59 on 14 df,   p=3e-10
Score (logrank) test = 74.49 on 14 df,   p=3e-10

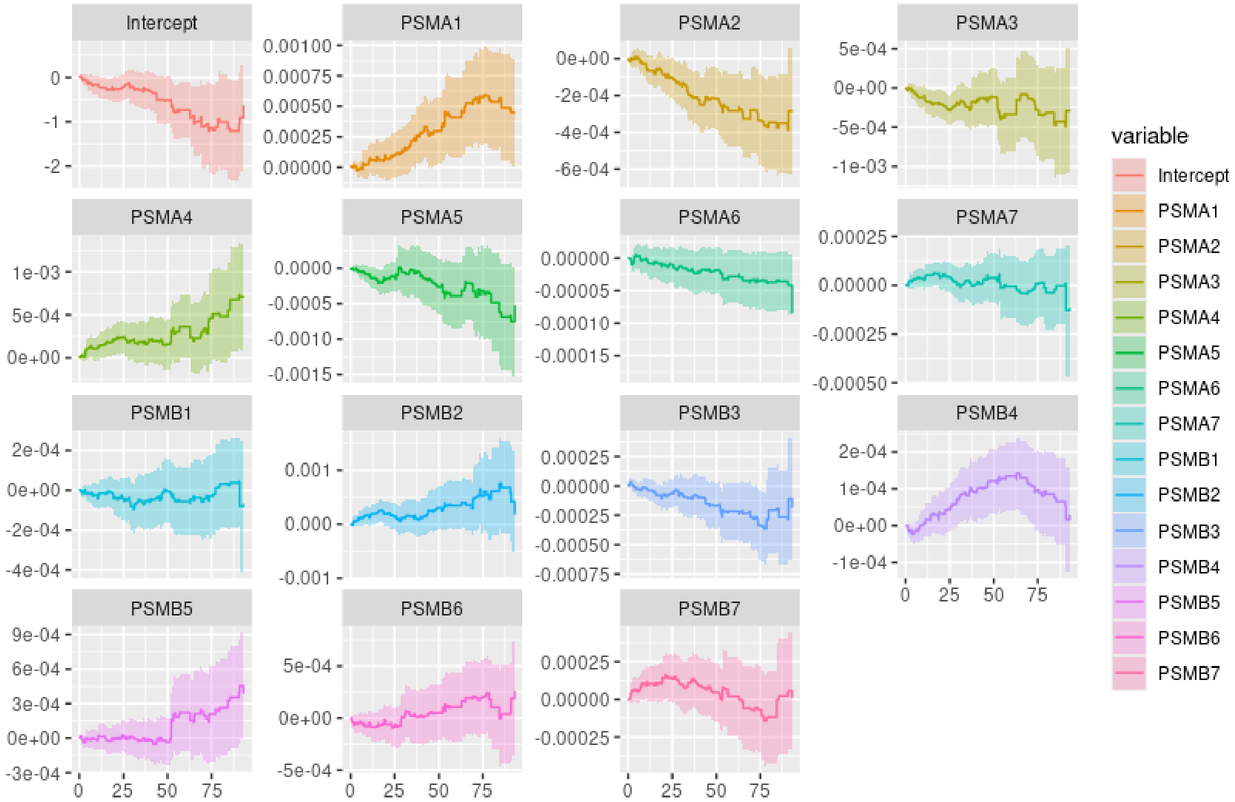

LGG

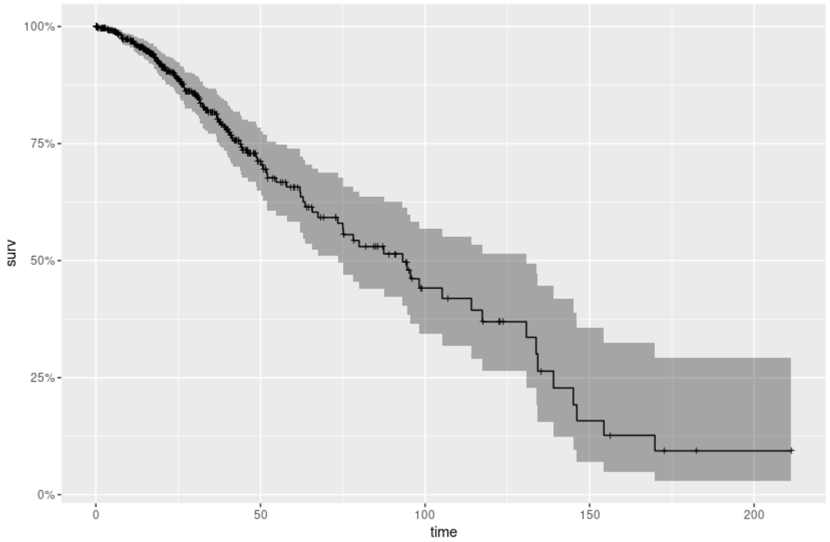

n= 513, number of events= 125
(1 observation deleted due to missingness)

	coef	exp(coef)	se(coef)	z	Pr(>|z|)	
PSMA1	0.0010421	1.0010427	0.0004544	2.293	0.02182	*
PSMA2	0.0006526	1.0006528	0.0003255	2.005	0.04500	*
PSMA3	-0.0016716	0.9983298	0.0008565	-1.952	0.05099	.
PSMA4	0.0003915	1.0003915	0.0004295	0.911	0.36205	
PSMA5	-0.0015567	0.9984445	0.0009127	-1.706	0.08810	.
PSMA6	-0.0002794	0.9997206	0.0004127	-0.677	0.49836	
PSMA7	0.0004898	1.0004899	0.0003211	1.526	0.12710	
PSMB1	0.0001639	1.0001639	0.0003169	0.517	0.60509	
PSMB2	0.0010713	1.0010719	0.0004407	2.431	0.01505	*
PSMB3	0.0006821	1.0006823	0.0005695	1.198	0.23102	
PSMB4	0.0001222	1.0001222	0.0001675	0.730	0.46568	
PSMB5	-0.0006019	0.9993983	0.0003131	-1.922	0.05458	.
PSMB6	-0.0008434	0.9991570	0.0006021	-1.401	0.16131	
PSMB7	-0.0009301	0.9990703	0.0003072	-3.028	0.00246	**

Signif. codes: 0 ‘***’ 0.001 ‘**’ 0.01 ‘*’ 0.05 ‘.’ 0.1 ‘ ’ 1

	exp(coef)	exp(-coef)	lower .95	upper .95
PSMA1	1.0010	0.9990	1.0002	1.0019
PSMA2	1.0007	0.9993	1.0000	1.0013
PSMA3	0.9983	1.0017	0.9967	1.0000
PSMA4	1.0004	0.9996	0.9995	1.0012
PSMA5	0.9984	1.0016	0.9967	1.0002
PSMA6	0.9997	1.0003	0.9989	1.0005
PSMA7	1.0005	0.9995	0.9999	1.0011
PSMB1	1.0002	0.9998	0.9995	1.0008
PSMB2	1.0011	0.9989	1.0002	1.0019
PSMB3	1.0007	0.9993	0.9996	1.0018
PSMB4	1.0001	0.9999	0.9998	1.0005
PSMB5	0.9994	1.0006	0.9988	1.0000
PSMB6	0.9992	1.0008	0.9980	1.0003
PSMB7	0.9991	1.0009	0.9985	0.9997

Concordance= 0.737 (se = 0.028)
Likelihood ratio test= 82.36 on 14 df, p=1e-11
Wald test = 89.96 on 14 df, p=4e-13
Score (logrank) test = 99.71 on 14 df, p=5e-15

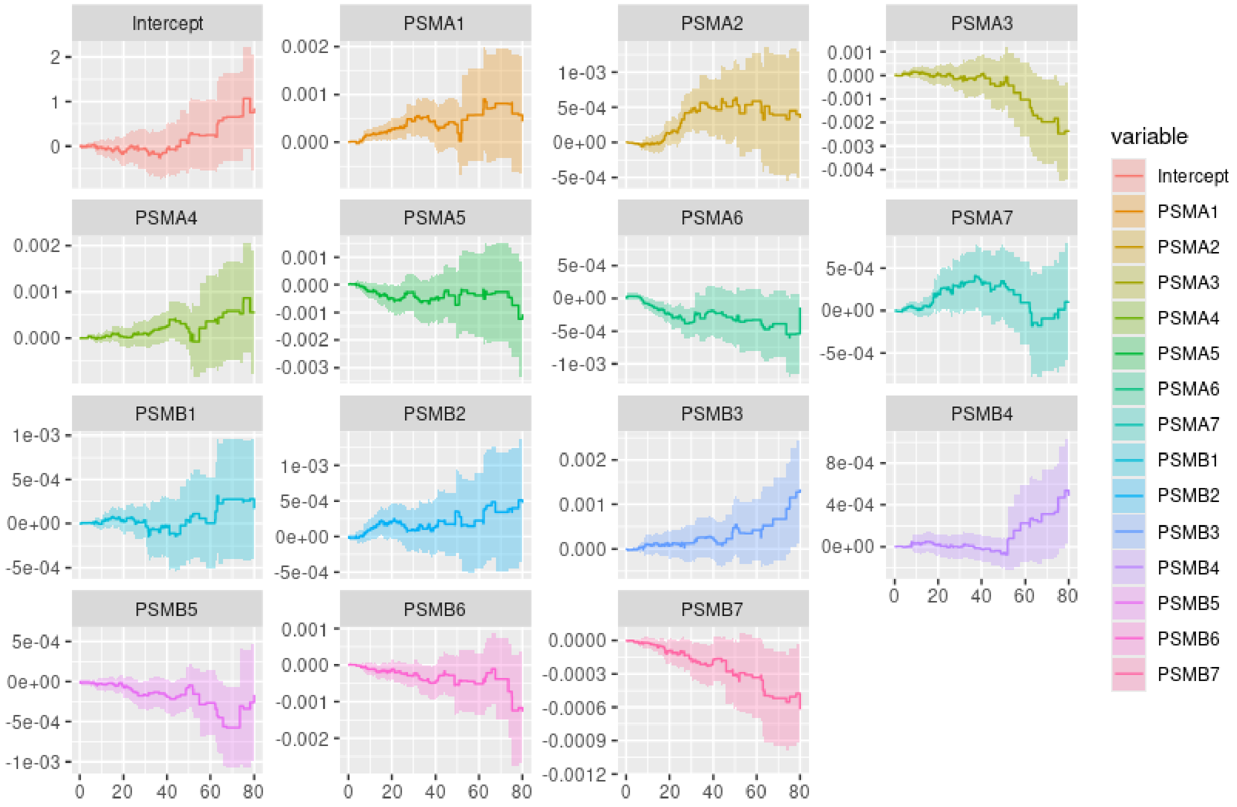

LUAD

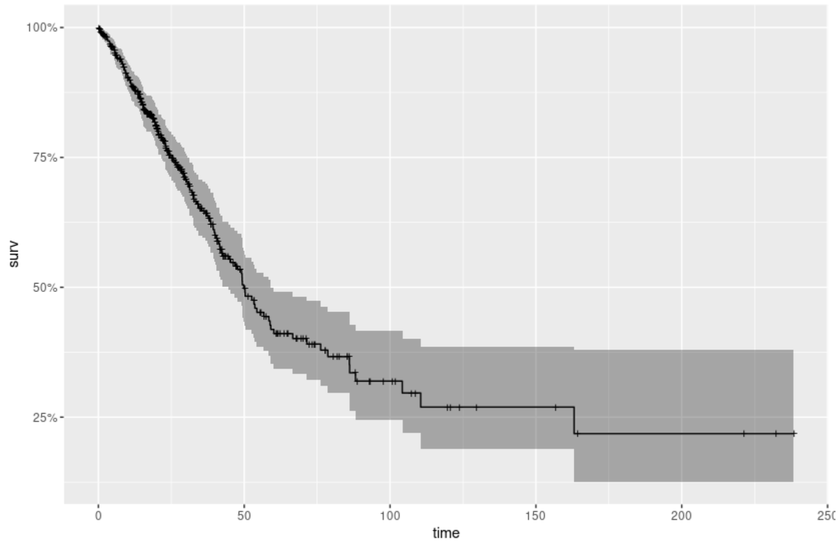

n= 501, number of events= 181
(9 observations deleted due to missingness)

	coef	exp(coef)	se(coef)	z	Pr(>|z|)	
PSMA1	-3.280e-05	1.000e+00	9.070e-05	-0.362	0.71759	
PSMA2	2.094e-04	1.000e+00	9.398e-05	2.228	0.02587	*
PSMA3	-5.826e-05	9.999e-01	1.314e-04	-0.443	0.65757	
PSMA4	9.546e-05	1.000e+00	1.016e-04	0.939	0.34763	
PSMA5	1.603e-04	1.000e+00	1.351e-04	1.186	0.23552	
PSMA6	-2.084e-05	1.000e+00	2.669e-05	-0.781	0.43496	
PSMA7	-1.014e-04	9.999e-01	6.578e-05	-1.541	0.12330	

```
PSMB1 -8.868e-05  9.999e-01  1.015e-04 -0.874  0.38236
PSMB2  1.033e-04  1.000e+00  9.616e-05  1.074  0.28284
PSMB3 -1.710e-04  9.998e-01  8.460e-05 -2.021  0.04324 *
PSMB4 -2.328e-05  1.000e+00  4.675e-05 -0.498  0.61853
PSMB5  1.031e-04  1.000e+00  5.601e-05  1.840  0.06576 .
PSMB6  3.526e-05  1.000e+00  1.216e-04  0.290  0.77174
PSMB7  3.056e-04  1.000e+00  1.129e-04  2.708  0.00678 **
---
Signif. codes:  0 '***' 0.001 '**' 0.01 '*' 0.05 '.' 0.1 ' ' 1

      exp(coef) exp(-coef) lower .95 upper .95
PSMA1    1.0000     1.0000    0.9998     1.000
PSMA2    1.0002     0.9998    1.0000     1.000
PSMA3    0.9999     1.0001    0.9997     1.000
PSMA4    1.0001     0.9999    0.9999     1.000
PSMA5    1.0002     0.9998    0.9999     1.000
PSMA6    1.0000     1.0000    0.9999     1.000
PSMA7    0.9999     1.0001    0.9998     1.000
PSMB1    0.9999     1.0001    0.9997     1.000
PSMB2    1.0001     0.9999    0.9999     1.000
PSMB3    0.9998     1.0002    0.9997     1.000
PSMB4    1.0000     1.0000    0.9999     1.000
PSMB5    1.0001     0.9999    1.0000     1.000
PSMB6    1.0000     1.0000    0.9998     1.000
PSMB7    1.0003     0.9997    1.0001     1.001

Concordance= 0.612  (se = 0.025 )
Likelihood ratio test= 33.94  on 14 df,   p=0.002
Wald test            = 38.5  on 14 df,   p=4e-04
Score (logrank) test = 38.03  on 14 df,   p=5e-04
```

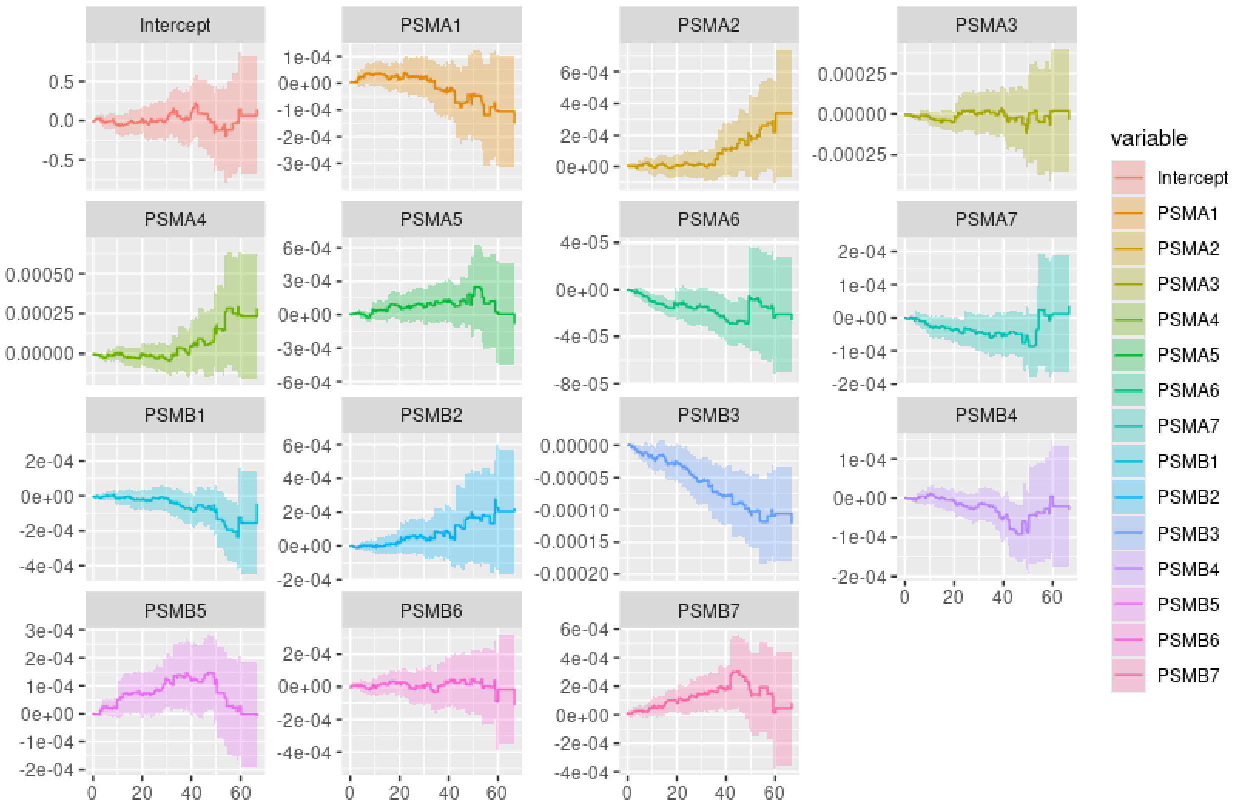

Supplement: Supplementary file 1 — Fig. S1. Electrostatic potential and surface hydrophobicity of the variants discovered in TCGA‐DU‐6392, visualized in ChimeraX. Fig. S2. Graphical summary from the IPA core analysis. Fig. S3. siRNA treatment of THP‐1 cells. Fig. S4. Cell viability assay after Bortezomib treatment in THP‐1 cells. Table S1. TCGA abbreviations and number of samples used in the survival study. Table S2. Proteins encoded by the proteasome subunits genes. Table S3. Primer sequences for qPCR. Table S4. Comprehensive variant evaluation and selection process. Table S5. Correlation between overall and proteasome mutations and Kaplan–Meier survival. Table S6. Identification of potentially disruptive variants in proteasome‐associated genes. Table S7. Differential expression factors of gene of interest by mean values. Table S8. Number of classified patients out of the whole cancer specific patient number. Table S9. 24 transporters of the ABC proteins family. Data S1. Data from TCGA and data from Cox‐regression analysis. [file MOL2-19-3729-s001.zip › MOLONC_25_0192_Supplementary data-Final_1.pdf]
