# Supplementary figures and images for: Expression and DNA methylation of 20S proteasome subunits as prognostic and resistance markers in cancer
Source: Mol Oncol. 2025 Aug 27;19(12):3729–49. doi: 10.1002/1878-0261.70038 (PMC12688171; doi:10.1002/1878-0261.70038)

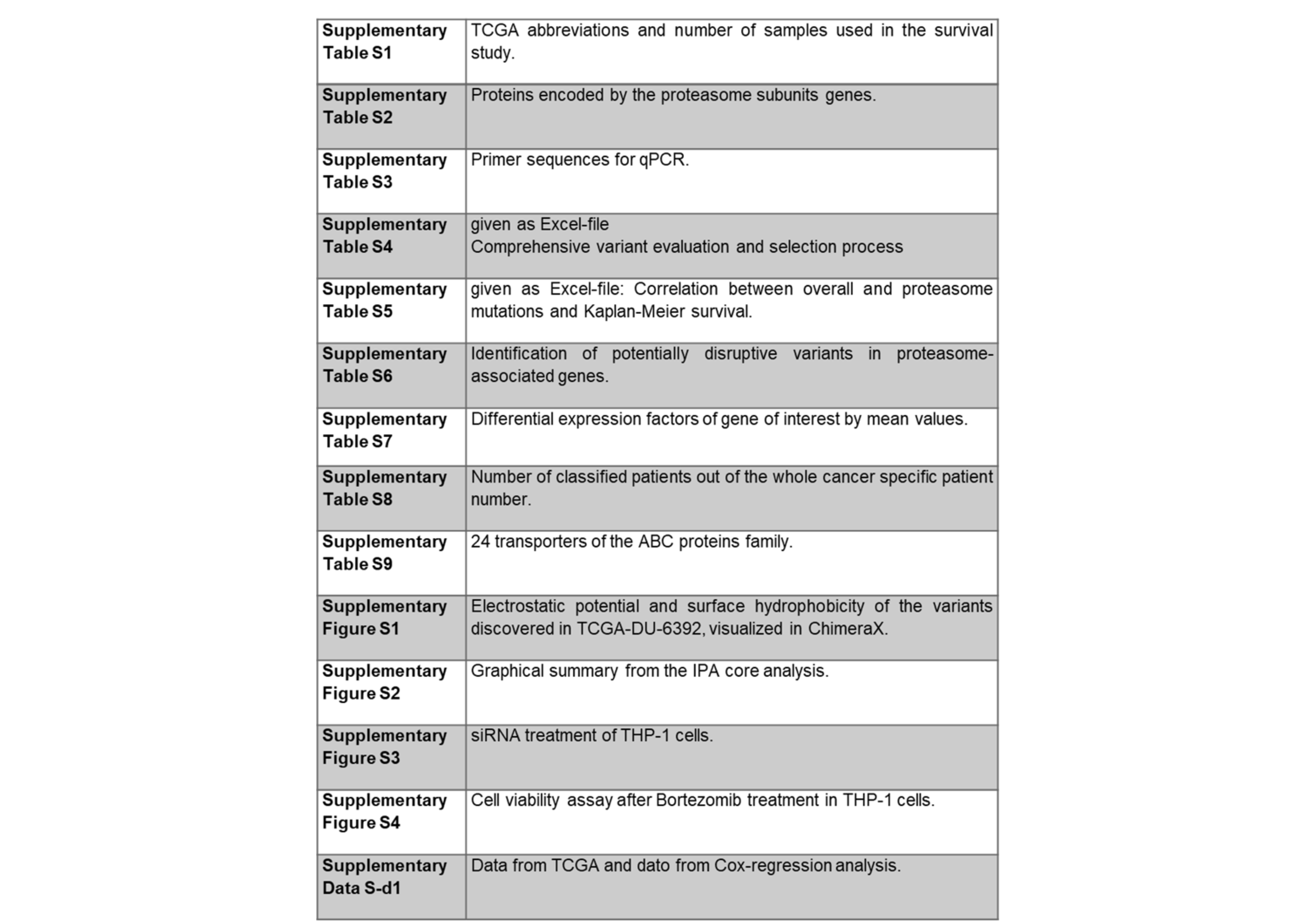

Supplement: Supplementary file 1 — Fig. S1. Electrostatic potential and surface hydrophobicity of the variants discovered in TCGA‐DU‐6392, visualized in ChimeraX. Fig. S2. Graphical summary from the IPA core analysis. Fig. S3. siRNA treatment of THP‐1 cells. Fig. S4. Cell viability assay after Bortezomib treatment in THP‐1 cells. Table S1. TCGA abbreviations and number of samples used in the survival study. Table S2. Proteins encoded by the proteasome subunits genes. Table S3. Primer sequences for qPCR. Table S4. Comprehensive variant evaluation and selection process. Table S5. Correlation between overall and proteasome mutations and Kaplan–Meier survival. Table S6. Identification of potentially disruptive variants in proteasome‐associated genes. Table S7. Differential expression factors of gene of interest by mean values. Table S8. Number of classified patients out of the whole cancer specific patient number. Table S9. 24 transporters of the ABC proteins family. Data S1. Data from TCGA and data from Cox‐regression analysis. [file MOL2-19-3729-s001.zip › TOC-immage-Supplementary.png]
